# Discovery of a deeply divergent new lineage of vine snake (Colubridae: Ahaetuliinae: *Proahaetulla* gen. nov.) from the southern Western Ghats of Peninsular India with a revised key for Ahaetuliinae

**DOI:** 10.1371/journal.pone.0218851

**Published:** 2019-07-17

**Authors:** Ashok Kumar Mallik, N. Srikanthan Achyuthan, Sumaithangi R. Ganesh, Saunak P. Pal, S. P. Vijayakumar, Kartik Shanker

**Affiliations:** 1 Center for Ecological Sciences, Indian Institute of Science, Bangalore, Karnataka, India; 2 Chennai Snake Park, Raj Bhavan post, Chennai, Tamil Nadu, India; 3 Bombay Natural History Society, Hornbill House, Opposite Lion Gate, Fort, Mumbai, India; 4 Department of Biological Sciences, The George Washington University, Washington, DC, United States of America; Centre for Cellular and Molecular Biology, INDIA

## Abstract

The Western Ghats are well known as a biodiversity hotspot, but the full extent of its snake diversity is yet to be uncovered. Here, we describe a new genus and species of vine snake ***Proahaetulla antiqua* gen. et sp. nov**., from the Agasthyamalai hills in the southern Western Ghats. It was found to be a member of the Ahaetuliinae clade, which currently comprises the arboreal snake genera *Ahaetulla*, *Dryophiops*, *Dendrelaphis* and *Chrysopelea*, distributed in South and Southeast Asia. *Proahaetulla* shows a sister relationship with all currently known taxa belonging to the genus *Ahaetulla*, and shares ancestry with *Dryophiops*. In addition to its phylogenetic position and significant genetic divergence, this new taxon is also different in morphology from members of Ahaetuliinae in a combination of characters, having 12–13 partially serrated keels on the dorsal scale rows, 20 maxillary teeth and 3 postocular scales. Divergence dating reveals that the new genus is ancient, dating back to the Mid-Oligocene, and is one of the oldest persisting monotypic lineages of snakes in the Western Ghats. This discovery adds to the growing list of ancient lineages endemic to the Agasthyamalai hills and underscores the biogeographic significance of this isolated massif in the southern Western Ghats.

## Introduction

The Western Ghats (WG) of Peninsular India is a global biodiversity hotspot with a high diversity of snakes [1–2]. While the mountain range has been explored since the colonial period, the systematics of snake fauna remains poorly known. There have been intensive explorations of WG biodiversity in the last decade revealing many new genera and species of trees [3–6], invertebrates [7,8], fish [9] and birds [10]. An increasing number of herpetological expeditions, and interest in the region, has also resulted in many discoveries [11–20]. This has steadily increased the importance of this biodiversity hotspot for conservation.

The vine snakes of Asia belong to the subfamily Ahaetuliinae within Colubridae [21]. Pyron et al. [21] first revealed the presence of a distinctive clade of arboreal, diurnal, tropical forest colubrids and recognized the subfamily Ahaetuliinae comprising genera *Dendrelaphis*, *Ahaetulla* and *Chrysopelea* using a large-scale molecular phylogeny with strong support for its monophyly. The generic term ‘vine snake’ is also used as a common epithet for snakes of the genera *Thamnodynastes*, *Oxybelis*, *Philodryas*, *Thelotornis* and *Xyelodontophis* (found in Africa and South America) due to their superficial visual characters such as a slender and sharp snout, slender bodies and a general vine like appearance.

Further studies [22, 23] corroborated earlier findings and expanded our understanding of Ahaetuliinae. This clade is split into two monophyletic groups, where one group consists of sharp-nosed snakes with a well-developed canthus rostralis and horizontal pupil currently represented by two genera–*Dryophiops* and *Ahaetulla*; and the other group consisting of rectangular snouted snakes with slightly compressed head and round pupils–*Dendrelaphis* and *Chrysopelea*. They also fixed earlier nomenclature issues such that their subfamilial nomen is conferred nomenclatural availability as per ICZN 1999. Currently, Ahaetuliinae comprises a total of 61 species belonging to four genera, *Ahaetulla* Link 1807 (9 species), *Chrysopelea* Boie 1826 (5 species), *Dendrelaphis* Boulenger 1890 (45 species) and *Dryophiops* Boulenger 1896 (2 species) which are mostly distributed in India and South East Asia. Of these four genera, *Ahaetulla* is widely distributed in Peninsular India and Sri Lanka as well as in Southeast Asia, while *Dryophiops* is distributed exclusively in Southeast Asia.

During the course of our work on the systematics and biogeography of snakes of Peninsular India [24], we collected a visually unusual vine snake from the Agasthyamalai hills in the southern Western Ghats. Due to its morphological differences from all recognized vine snakes from that region, we further investigated its systematic position. Our molecular phylogenetic analyses revealed that the unknown vine snake shares a common ancestor with the genus *Ahaetulla* (South + SE Asian), and the clade composed of *Ahaetulla* and the new vine snake as sister to the Malayan *Dryophiops*. In this communication, we formally name and describe this taxon as a new genus and species, with an emphasis on the phylogenetic relationship and divergence time within the subfamily Ahaetuliinae.

## Methods

We collected two individuals of the new taxon from close to Agasthiyar peak (8°37'09"N 77°14'57E) in Kalakkad Mundanthurai Tiger Reserve and Pandimotta (8°49'35N 77°13'02E) in Shendurney Wildlife Sanctuary in Agasthyamalai, the southern most hill range in the Western Ghats. The individuals were euthanized using Diethyl ether as anesthetic agent before preservation. Individuals were fixed using 70% alcohol. Liver tissue was collected and preserved in 95% alcohol. The specimens were deposited in the museum collection of the Center of Ecological Sciences (CES), Indian Institute of Science, Bangalore, India. A total of 14 specimens of *Ahaetulla* spp. representing the thus far known Peninsular Indian congeners (*A*. cf. *nasuta*, *A*. *dispar*, *A*. cf. *pulverulenta*, *A*. *perroteti* and *A*. *prasina*) [25] were examined as comparative material for this study (S1 Appendix & S2 Table). The necessary biological specimen collection permits were acquired from the state forest departments of Tamil Nadu, Kerala, Karnataka, Maharashtra and Arunachal Pradesh.

We also used comparative data from published sources for four more congeners of *Ahaetulla*, namely *A*. *anomala* from Peninsular India, and *A*. *fronticincta*, *A*. *mycterizans* and *A*. *fasciolata* from Southeast Asia, as well as from representative members of all other genera in the clade–*Dendrelaphis* (*D*. *tristis* (4), *D*. *cyanochloris*, *D*. cf. *pictus*, *D*. cf. *ashoki*, *D*. cf. *chairecaeos*, *D*. cf *girii* & *Dendrelaphis* sp.), *Chrysopelea* (*C*. *ornate*, C. *taprobanica* (2) & *C*. cf. *taprobanica*) and *Dryophiops* (*D*. *philippina & D*. *rubescens*) (S2 Table) [25–28].

### Molecular analysis

#### DNA extraction and amplification

We extracted total genomic DNA from new taxon voucher specimens (CESS 259 and CESS 318 tissue samples stored in 95% absolute alcohol). Genomic DNA was extracted using a commercially available DNEasy extraction kit (QIAgen). Three mitochondrial genes, Cytochrome-b (Cytb, 1048 bp), NADH dehydrogenase subunit 4 (ND4, 663 bp), 16S rRNA (472 bp) and two nuclear genes, Oocyte maturation factor (c-*mos*, 552 bp) and recombination activating gene 1 (RAG1, 855 bp) were amplified with previously published primers (S1 Table). The PCR conditions and preparation protocol were the same as earlier studies [29–32]; however minor modifications of annealing temperature were applied in a few PCR reactions. The PCR amplified products were purified using QIAquick PCR purification kit (Qiagen). The cycle sequencing of purified products was carried out commercially at the Centre for Cellular and Molecular Platforms (C-CAMP, NCBS), Bangalore, India.

#### Sequence alignment

Sequences were edited and visually corrected using MEGA v5.2 [33]. The individual consensus sequences were derived from forward and reverse complements after checking for base mis-calls. The sequence alignment was accomplished using MUSCLE [34] implemented in MEGA v5.2. The protein coding genes (Cytb and ND4) were checked for the presence of indels and noncoding sequences to detect possible pseudogene amplification and premature stop codons by translating DNA to protein. No indels were detected in Cytb, ND4, c-*mos* and RAG1. We downloaded all available sequences of five genes for *Ahaetulla*, *Dryophiops*, *Chrysopelea* and *Dendrelaphis* from GenBank, but we could not use a few RAG1 sequences from other studies [23] in the final analyses due to their origin from a different section of the RAG1 gene. 16S rRNA sequences were aligned against available data in GenBank submitted by earlier studies and visually edited. Ambiguously aligned regions along with gaps were cropped out of the analysis due to the presence of secondary structures in the 16S rRNA sequence [35–37]. A concatenated dataset of 3590 bp was created combining all five data sets. The gaps present in the dataset were treated as missing data.

Outgroup sequences of the genera *Hemorrhois*, *Eirenis*, *Dolichophis*, *Zamenis*, *Lycodon*, *Oligodon*, *Xenochrophis*, *Rhabdophis*, *Natrix*, *Opisthotropis*, *Naja*, *Ophiophagus*, *Lycophidion*, *Duberria*, *Leioheterodon*, *Psammophis*, *Xenodermus*, *Sistrurus*, *Crotalus*, *Bothrops*, *Daboia*, and *Echis* were obtained from GenBank (S2 Appendix).

#### Phylogenetic analysis

We used PartitionFinder v1.1.1 [38] to identify suitable partitions in the datasets and the respective substitution model for each partitioned subset. The best-fit partition scheme was used for data partitioning prior to analysis (Table 1). Phylogenetic reconstructions were carried out using the maximum likelihood (ML) method. The Maximum likelihood tree was then reconstructed with non-parametric bootstrapping in raxmlGUI v1.3 [39, 40]. The RAxML platform implements only the GTR substitution model. The ML analysis was carried out with 1000 bootstrap replicates and GTR-GAMMA model was applied to every partition.

**Table 1:** The best-fit partition schemes used for Bayesian inference (MrBayes) and divergence dating (BEAST) predicted by PartitionFinder v1.1.1. The predicted best-fit models are the same for both analyses except for partition no. 6 where it is TrN+G for BEAST. Cp1- cp3 indicates the codon positions of each locus.

Partition no.	Partition	Best fit model
1	Cytb-cp1	GTR+I+G
2	Cytb-cp2 + ND4-cp2	GTR+I+G
3	Cytb-cp3 + ND4-cp3	GTR+G
4	ND4-cp1	GTR+I+G
5	C*-mos*-cp1 + RAG1-cp1	GTR+G
6	C*-mos*-cp2	HKY+G
7	C*-mos*-cp3	GTR+G
8	RAG1-cp2	HKY+I
9	RAG1-cp3	GTR+G
10	16S	GTR+I+G

Bayesian inference analysis was carried out using MrBayes v3.2 [41] and implemented in the online-based server CIPRES Science Gateway [42]. The analyses were carried out with the respective substitution models for each partition, with two parallel runs with four chains, temperature as 0.2, for 50 million generations sampling every 5000 generations from each analysis. Other parameters were set to the default settings. The analysis was terminated once it reached the standard deviation of the split of frequencies 0.01. We diagnosed the state of convergence of chains and ensured that the effective sample size (ESS) was above 200 for each parameter in Tracer v1.6 [43]. Additionally, log likelihood scores were plotted against other parameter values from each run against generation time. A final consensus tree and clade posterior probabilities were summarized after 25% of burn-in of the total number of trees sampled. The resulting ML and BI trees topologies were visualized in FigTree v1.4.2 [44].

#### Divergence dating

The divergence time was estimated with the DNA dataset used for Bayesian inference and maximum likelihood analysis and based on four fossil and biogeographic reference calibration points in BEAST v1.8.2 [45, 46]. We used the Birth-Death process for the tree prior, which is a continuous-time Markov process of speciation and extinction through time from an initial lineage to the birth of a new lineage [47]. We used an uncorrelated relaxed clock model with lognormal distribution [48] and GTRGAMMAI model for all the partitions in BEAST [40, 49]. The substitution models and tree topology were linked and clock model was unlinked among partitions (Table 1).

Three external fossil records [50–54] and a secondary calibration [49] were placed on the nodes after carefully considering the calibrations as suggested in an earlier study [49] (S3 Table). This Bayesian analysis was carried out twice using MCMC algorithm and 50 million generations sampled every 10000 generations and implemented in the online-based server CIPRES Science Gateway [42]. The convergence and effective estimated sample size (ESS) of the posterior probability distribution were estimated for all parameters in Tracer v1.6.2 [43]. 25% of the trees were discarded and the final divergence estimate tree was derived using a maximum clade credibility tree and node height kept as median in TreeAnnotator 1.8.2 [45, 46]. Trees were visualized in FigTree 1.4.2 [44].

### Morphological data

Snout to vent length, tail length and total body length were measured by marking with a string and using a measuring tape. Ventral scales were counted according to Dowling’s scheme [55]. Subcaudals were counted on one side excluding the terminal scale. Measurements were taken using Mitutoyo dial calipers. Meristic characters that were collected from the specimens included: pre-ventrals, ventrals (V), subcaudals (SC), supra-labials–right (SLr), supra-labials–left (SLl), largest supra-labial, supra-labials in contact with the eye–right, (SL2r), supra-labials in contact with the eye–left (SL2l), loreal–right (Lr), loreal–left (Ll), nasal–right, nasal–left, pre-subocular–right (PRSOr), pre-subocular–left (PRSOl), pre-ocular–right, pre-ocular–left, infra-labials–right (ILr), infra-labials–left (ILl), post-ocular–right (POr), post-ocular–left (POl), gulars–right, gulars–left, scales around the body (N-after neck, M-at midbody and T-before vent), temporals–right, temporals–left, sub-ocular–right, sub-ocular–left, pre-frontal and pre-ocular contact, cloacal plate, supra-labial scale division if any and the nature of ventrals (angulated, keeled, notched, no ventral keels). The mensural characters that were collected from the specimens are as follows: snout to vent length, tail length, total length, head length, nostril to eye length, vertical eye diameter, horizontal eye diameter, eye to snout length, pre-frontal length, frontal length, snout to vent length divided by the tail length, snout to vent length divided by head length and the relative tail length. Coloration characteristics that were considered are as follows: dorsal coloration, inter-scalar skin colour, cross bars across the body, iris colouration, ventral colour and ventral stripes. Hemipenis was everted and examined in situ. The upper jaws of the specimens were carefully dissected for counting the maxillary teeth. Empty sockets when present were counted as proxies for teeth and not as diastema.

#### Morphological analyses

A principle component analysis (PCA) was performed separately using all the characters to assess the major loading components. These contributing morphological characters were plotted using a multivariate analysis (V, SC, SL, SL2, L, PRSO, IL, PO, M and keels) that exhibited variability. We tested for differences in morphological space by comparing taxa representing all genera of the subfamily Ahaetuliinae with our primary and secondary datasets (S2 Table). The statistical analysis was carried out using PAST v3.14 for Macintosh [56].

### Nomenclatural acts

The electronic edition of this article conforms to the requirements of the amended International Code of Zoological Nomenclature, and hence the new names contained herein are available under that Code from the electronic edition of this article. This published work and the nomenclatural acts it contains have been registered in ZooBank, the online registration system for the ICZN. The ZooBank LSIDs (Life Science Identifiers) can be resolved and the associated information viewed through any standard web browser by appending the LSID to the prefix "http://zoobank.org/". The LSID for this publication is: urn:lsid:zoobank.org:pub: 777BD774-53BE-432D-AF2F-3948B8E1E8D4. The electronic edition of this work was published in a journal with an ISSN, and has been archived and is available from the following digital repositories: PubMed Central, LOCKSS.

## Results

### Phylogenetic relationship of Ahaetuliinae

**Fig 1:**
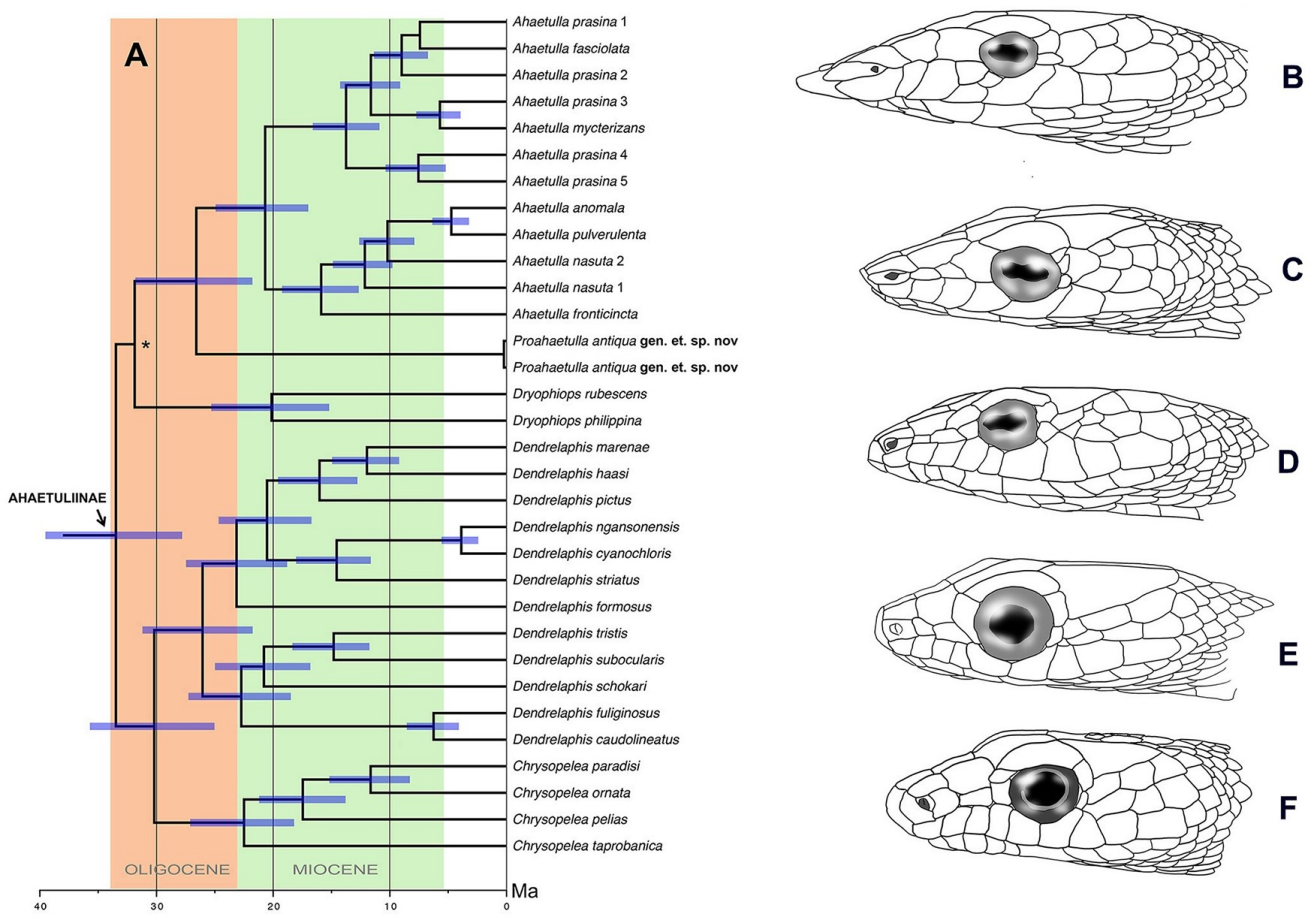
Phylogenetic relationship of *Proahaetulla antiqua* gen. et sp. nov. **(A)** Chronogram shows phylogenetic relationship and time of divergence of *Proahaetulla antiqua* **gen. et sp. nov**. within family Ahaetuliinae. Asterisk (*) indicates lower posterior probability support on the node from Bayesian inference. Bar on each node indicates 95% HPD. (B) Head profile of *Ahaetulla*, (C) *Proahaetulla* **gen. nov**., (D) *Dryophiops*, (E) *Dendrelaphis* and (E) *Chrysopelea*.

**Fig 2:**
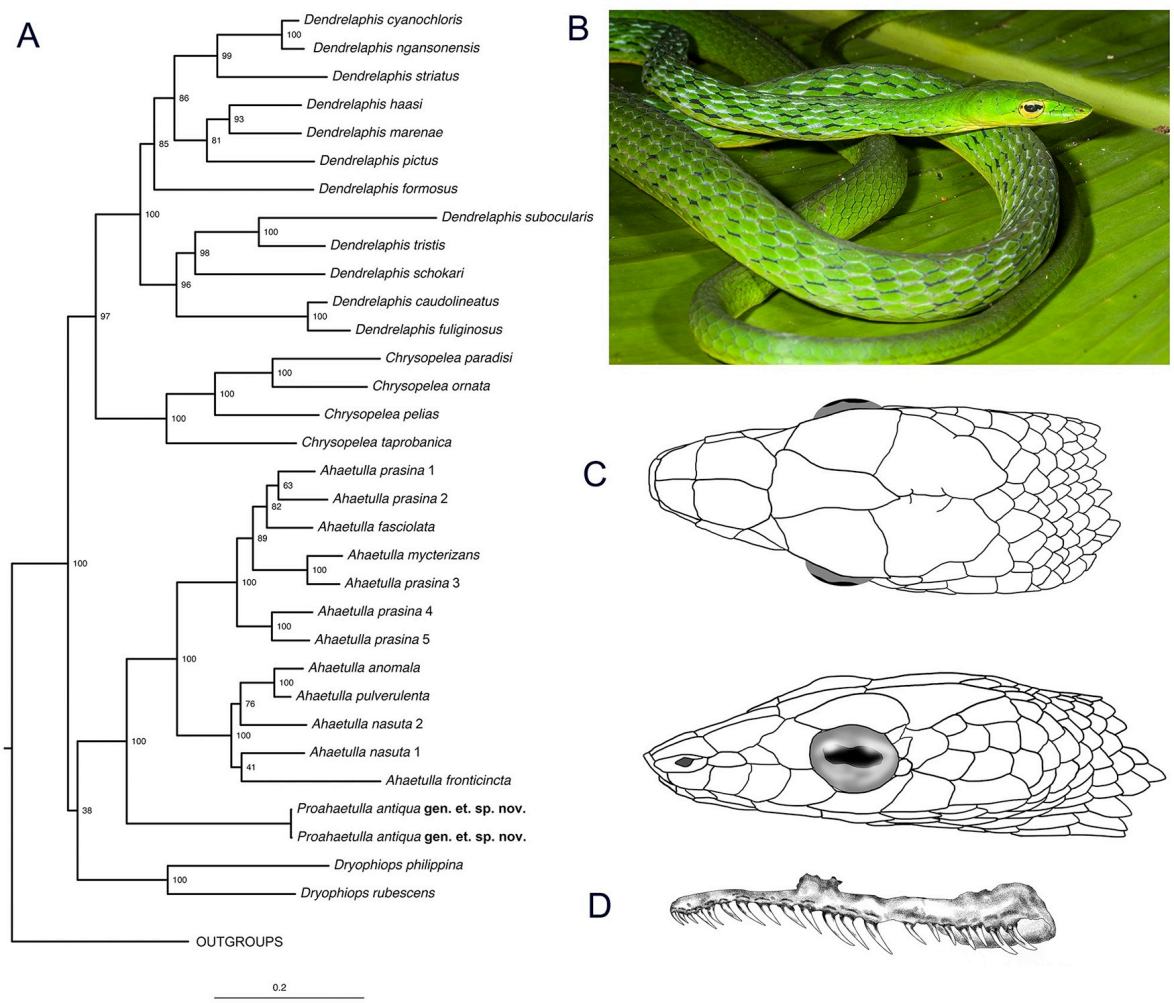
(A) **Maximum likelihood tree showing the relationship of *Proahaetulla antiqua* gen. et sp. nov. within family Ahaetuliinae**. Value on each node indicates the bootstrap support. (B) Photograph of holotype specimen in life. (C) Dorsal and lateral view of head. (D) Dentition arrangement of maxillary arch.

The tree topology recovered from a Bayesian analysis for Ahaetuliinae (comprising currently known genera) was similar to the result obtained by an earlier study [23]. We found that ***Proahaetulla antiqua* gen. et sp. nov**. is nested within the Ahaetuliinae clade and shares ancestry (PP 1.0 & ML bootstrap >70%) with the rest of the members of genus *Ahaetulla* (Fig 1A). The clade comprising *Ahaetulla* and ***Proahaetulla antiqua* gen. et sp. nov**. was recovered as sister to the *Dryophiops* clade but with low bootstrap support (< 70%); however, the relationship between *Ahaetulla* and *Dryophiops* is strongly supported in a previous study [23] (Fig 2).

There is 14.1–17.4% (on Cytb), 16.0–18.1% (on ND4) and 5.2–6.5% (on 16S) genetic divergence between the new lineage *Proahaetulla* **gen nov**. and *Ahaetulla* (Table 2). This distance is comparable with the genetic divergence value between two other related sympatric genera *Chrysopelea* and *Dendrelaphis* (13.7–22.0% on Cytb, 15.3–20.1% on ND4 and 4.1–8.2% on 16S). Moreover, the genetic distance between *Proahaetulla* **gen nov**. and *Ahaetulla* (on 16S) is in the same range as the distance between *Dryophiops* and *Ahaetulla*, which are also distributed sympatrically. Given the genetic divergence between these established genera in the clade Ahaetuliinae, there is a strong case to treat *Proahaetulla* as a genus based on genetic distance alone.

**Table 2:** Genetic distances *p*-between members of Ahaetuliinae.

Gene		Genus	1	2	3	4
**Cytb**	**1**	*Proahaetulla*				
	**2**	*Ahaetulla*	14.1–17.4			
	**3**	*Dryophiops*	16.6–20.3	16.4–21.2		
	**4**	*Dendrelaphis*	16.6–21.6	12.7–22.3	14.3–21.2	
	**5**	*Chrysopelea*	13.4–18.9	13.0–18.9	16.5–20.8	13.7–22.0
**ND4**	**1**	*Proahaetulla*				
	**2**	*Ahaetulla*	16.0–18.1			
	**3**	*Dryophiops*	17.4–17.5	18.0–19.6		
	**4**	*Dendrelaphis*	17.4–21.3	16.8–22.2	16.8–21.1	
	**5**	*Chrysopelea*	17.5–18.9	16.0–20.5	16.8–18.4	15.3–20.1
**16S**	**1**	*Proahaetulla*				
	**2**	*Ahaetulla*	5.2–6.5			
	**3**	*Dryophiops*	6.0–6.7	5.2–6.7		
	**4**	*Dendrelaphis*	6.5–8.1	4.5–8.7	5.4–7.2	
	**5**	*Chrysopelea*	6.9–7.8	5.0–7.8	5.2–6.9	4.1–8.2

### Time of divergence

The MRCA of family Ahaetuliinae dates to 33.63 Ma (HPD 27.78–39.43). The MRCA of genus *Ahaetulla* and *Proahaetulla antiqua* **gen. et sp. nov**. is dated at 26.55 Ma (HPD 21.36–31.50), which suggests that *Proahaetulla antiqua* **gen. et sp. nov**. is an old, deeply divergent lineage within this clade (Fig 1A).

### Morphology

**Fig 3:**
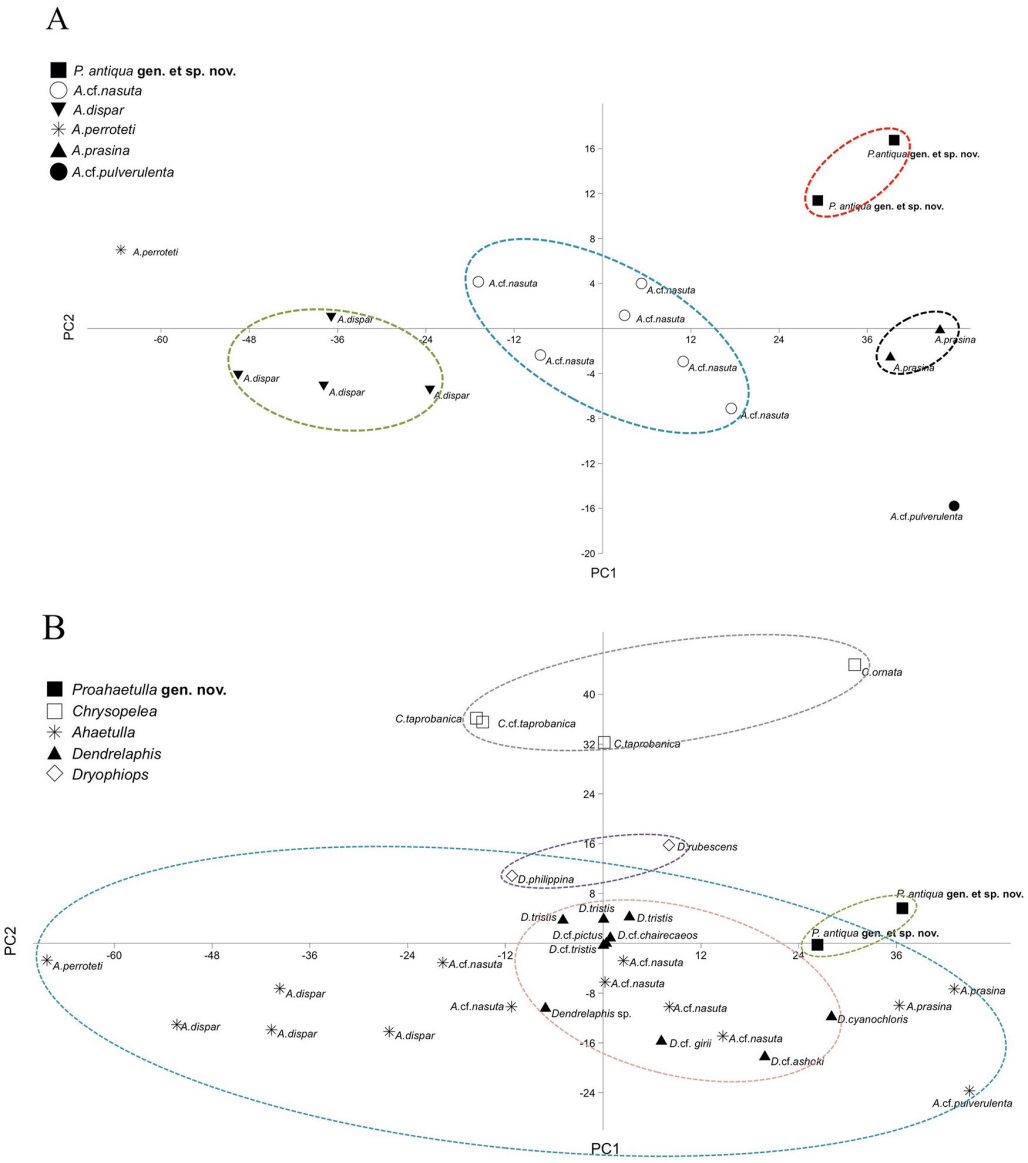
Morphological space shared by *Proahaetulla* gen. nov. and all other genera of the subfamily Ahaetuliinae. (A) A PCA showing morphological space shared by *Ahaetulla* and *Proahaetulla* **gen nov**. in Peninsular India. (B) A PCA of members (all five genera) of Ahaetuliinae shows the overlapping morphological space across the subfamily.

A principal component analysis including members of *Ahaetulla* from Peninsular India and *Proahaetulla antiqua* **gen. et sp. nov**. indicated that *Proahaetulla antiqua* **gen. et sp. nov**. occupies a separate morphological space on the PCA plot (Fig 3A and S4 Table). PC1 and PC2 explained 94.6% and 4.4% variance respectively. The number of sub-caudal scales had a higher loading (0.82) on PC1 while ventral scales had a higher loading (0.72) on PC2.

We also carried out a PCA including taxa representing all genera of the subfamily Ahaetuliinae (Fig 3B and S4 Table). Here, PC1 and PC2 explained 70.1% and 27.3% of the variance; the number of sub-caudal scales had a higher loading (0.81) on PC1 while ventral scales had a higher loading (0.81) on PC2. There is overlap in the morphological space occupied by *Ahaetulla*, *Proahaetulla* **gen. nov**., *Dryophiops* and *Dendrelaphis*. *Dryophiops* and *Proahaetulla* **gen. nov**. overlap peripherally (for known taxa) while *Dendrelaphis* is nested within *Ahaetulla*.

*Ahaetulla* occupies a wide morphological space in the plot due to a high level of morphological divergence between different species, which display variation in Ventrals (140–200), Caudals (84–189), Loreals (0–2), Post-Ocular (1–2), and Keels (0–2) within Peninsular Indian congeners. The morphological space of the genus *Ahaetulla* as a whole is even greater as taxa from other regions show variation beyond this range (i.e. V 211–240 & C 178–197 in *Ahaetulla fasciolata*, and V 182–203 C 169–208 in *Ahaetulla pulverulenta*).

The other genera (*Dendrelaphis* from India, *Chrysopelea*, *Dryophiops* and *Proahaetulla*) occupy a relatively small morphological space compared to *Ahaetulla*. However, *Dendrelaphis* has 45 species across its range, and it is expected that the morphological space occupied by this genus would be much larger if all the taxa were included in the analysis.

*Chrysopelea* occupies a non-overlapping position with respect to the other genera due to the variation in a few morphological characters (V 201–236, C 106–138 and M 17), but this does not represent the full range of variation in the genus. The morphological space of *Ahaetulla* overlaps with *Dryophiops*, *Dendrelaphis* and *Proahaeatulla* **gen. nov**. due to the presence of several synapomorphic morphological characters (L, PO, PRSO, M and keels) although there are many diagnostic morphological characters (V, C, SL, IL, PO and keels).

### Systematics

Ahaetuliinae Figueroa, McKelvy, Grismer, Bell, Lailvaux, 2016

Ahaetuliinae–Pyron, Burbrink, Wiens, 2013 (invalid nomen).

Ahaetullinae–Zheng & Wiens, 2015 (invalid nomen).

*Proahaetulla* **gen. nov**.

urn:lsid:zoobank.org:act:E1A80BE5-F261-4148-BDEA-E48B2B94A048

**Type species**, by present designation: *Proahaetulla antiqua* **sp. nov**., by monotypy.

urn:lsid:zoobank.org:act:D1723AB6-27FE-4EF1-BE03-D43B2EF9EC55

**Holotype**: CESS259; adult male; near Agasthiyar peak (8°37'09''N 77°14'57''E), Agasthyamalai hills, Kalakad Mundanthurai tiger reserve, Tamil Nadu, India; *Coll*. Saunak Pal and S. P. Vijayakumar, 28^th^ August 2011.

**Paratype**: CESS318; adult male; Pandimotta (8°49'35''N 77°13'02''E), Shendurney Wildlife Sanctuary, Thenmala, Kerala, India; *Coll*. S.R. Chandramouli and K. P. Dinesh, July 2012.

**Etymology**: The generic epithet *Proahaetulla* **gen. nov**. stems from the generic nomen *Ahaetulla* indicating the early divergence of the lineage from the rest of the Ahaetuliinae members. Gender feminine. The specific epithet *antiqua* is Latin for ‘antique’ or old, a term alluding to the evolutionary age or antiquity of this new taxon.

### Diagnosis

#### Lineage diagnosis

1. ***Proahaetulla* gen. nov**. is a member of the subfamily Ahaetuliinae, and shares ancestry and a sister relationship with *Aheatulla* clade.
2. It shows high genetic divergence and differs from members of the genus *Ahaetulla* (including *A*. *nasuta*, *A*. *pulverulenta*, *A*. *prasina*, *A*. *anomala*, *A*. *fronticinta*, *A*. *fasciolata* and *A*. *mycterizans)* with genetic distances of 14.1–17.4% on Cytb, 16.0–18.1% on ND4 and 5.2–6.5% on 16S genes.
3. Morphologically, it is characterized by the presence of horizontally elliptical pupil; concave loreal region, enabling a near-binocular vision; snout produced forward to a fine point; and bright-green dorsum. ***Proahaetulla* gen. nov**. differs from *Ahaetulla* (*A*. *anomala*, *A*. *nasuta*, *A*. *dispar*, *A*. *pulverulenta*, *A*. *mycterizans*, *A*. *fasciolata*, *A*. *fronticincta* & *A*. *prasina*) in showing the following combination of morphological characters: 12–13 rows of mildly serrated, keeled dorsal scales starting from the nape till the dorsal scales that are situated in the row adjacent to the cloacal plate; strongest keels on the mid vertebral row (7^th^ or 8^th^ row) of scales; keels consecutively weaker in the paravertebral row of scales (dorsal scales smooth in most *Ahaetulla*, partly keeled near sacral region in *A*. *perroteti*); a set of 20 maxillary teeth (12–16 in *Ahaetulla*) and post-ocular scales 2–3 (2 in *Ahaetulla*); greenish yellow tongue with black speckles (vs. reddish, purplish or brownish tongue in *Ahaetulla* spp.).
4. ***Proahaetulla* gen. nov**. differs from all the known genera in Ahaetuliinae as follows: pupil horizontally elliptical (vs. rounded in *Dendrelaphis*, *Chrysopelea*); snout tip pointed (vs. rounded in *Dendrelaphis*, *Chrysopelea*); dorsum verdant green (vs. never totally green in *Dendrelaphis*, *Chrysopelea*, *Dryophiops*); dorsal scales keeled (vs. smooth in *Dendrelaphis*, *Dryophiops*, *Ahaetulla*); vertebral scales not enlarged (vs. enlarged in *Dendrelaphis*); dorsal body scales without apical pits (vs. with apical pits in *Dendrelaphis*, *Chrysopelea*, *Dryophiops*); maxillary teeth 20 (vs. < 16 in Ahaetulla; > 22 in *Dendrelaphis*, *Chrysopelea*); mid body scale rows 13–15 (vs. not less than 15 in *Dryophiops*, *Ahaetulla*; 17 in *Chrysopelea*) (Table 3).

**Table 3:** Comparison of morphological characters of members of the subfamily Ahaetuliinae.

Genus	*Dendrelaphis*	*Chrysopelea*	*Dryophiops*	*Ahaetulla*	*Proahaetulla* gen. nov.
Dorsal scales keeled	Present	Present	Present	Absent	Present
Oblique Dorsal Scales	Present	Present	Present	Present	Present
Maxillary tooth	20 to 34	22 to 22	20	12 to 16	20
Pupil shape	Round	Round	Horizontal	Horizontal	Horizontal
Sharp snout	Absent	Absent	Present	Present	Present
Ventral Keels	Present	Present	Present	Present	Present
Dorsal keels	Absent	Present	Absent	Absent	Present
Apical pits	Present	Present	Present	Absent	Absent
Ventrals	149 to 213	181 to 236	177 to 199	136 to 240	196 to 207
Subcaudals	74 to 175	89 to 180	111 to 136	65 to 208	160 to 165
Scale rows	13 to 15	17	15	15	13 to 15
Post oculars (R+L)	1 to 3	2	2 to 3	1 to 2	2 to 3

**Description of Holotype**: Adult male of total length 1113 mm; hemipenis reversed, dissected; very slender and partially laterally compressed body with snout to vent length 702 mm; tail relatively long and slender with length 411 mm; relative tail length 0.37; ventrals 196, notched with keels; subcaudals 160, divided; cloacal scale divided; scale rows 15-15-13 (after neck, at midbody and before vent); 12 rows of mildly serrated, keeled dorsal scales starting from the nape till the dorsal scales that are situated in the row adjacent to the cloacal plate; last row of scales in contact with the ventrals smooth; strongest keels on the mid vertebral row (8^th^ row) of scales; keels consecutively weaker in the paravertebral row of scales; head very distinct from neck with head length 22.5mm; transversely oval eyes with horizontal pupil; horizontal diameter of the eye 4.5 mm and vertical diameter of the eye 3.6 mm; distance from nostril to eye 5.8 mm; distance from snout tip to eye 8.1 mm; supralabials 7 (both left and right) with the 4^th^ and 5^th^ supralabial touching the eye and the 6^th^ supralabial being the largest; no visible supralabial scale division; 8 infralabials (both left and right), 2^nd^, 3^rd^ and 4^th^ infralabials in contact with the anterior genials; 4^th^ and 5^th^ infralabials in contact with the posterior genials; mental scale wedged in between 1^st^ pair of infralabials not in contact with the genials; single nasal scale (both left and right); two loreals on each side (both left and right); pre-suboculars absent; single pre-ocular (both left and right); post-oculars two in the right and three in the left; sub-oculars absent; temporals 1+3 (anterior + posterior) on the right and 2+3 (anterior + posterior) on the left; prefrontal scale in contact with the preoculars; two preventrals (Fig 2B–2D).

**Colour in life**: Body uniform bright green (Fig 2B); rostral, infralabials and mid body on the underside creamish yellow to light green; creamish yellow ventral stripe along the notched ventral keels; slight discoloration in the preocular; when threatened, the inter-scalar skin is revealed, colored with a consecutive series of black and white bars that converge towards the head; an occasional light blue coloration at the proximal end of scales; concentration of black speckles both in the anterior and posterior end of the horizontal pupil and a slight discoloration around the pupil; tongue greenish yellow.

**Fig 4:**
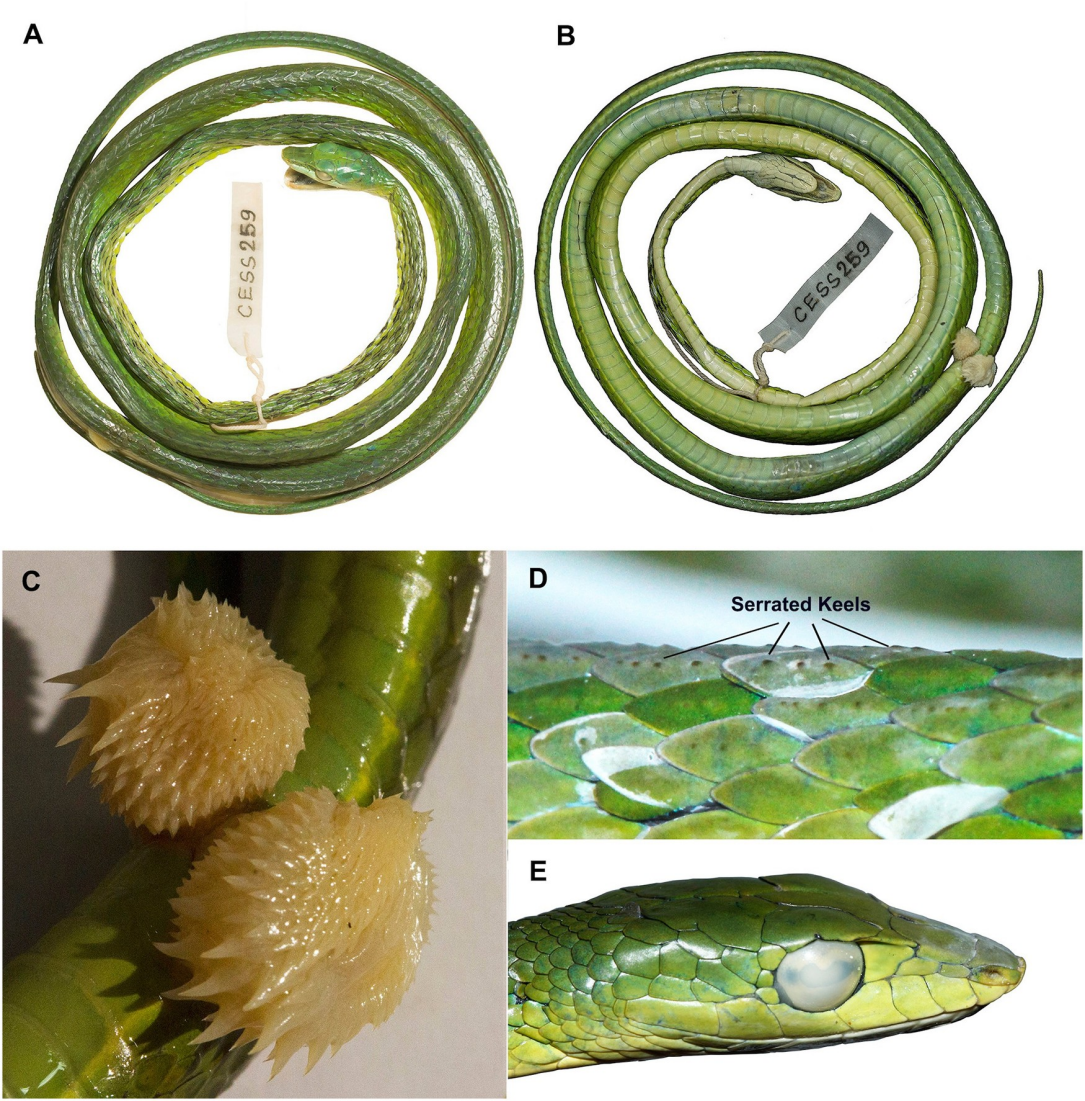
*Proahaetulla antiqua* gen et sp. nov. holotype (CESS 259). (**A**) Dorsal view. (**B**) Ventral view. (**C**) Hemipenal profile. (**D**) Dorsal scales showing serrated keels. (**E**) Lateral view of head of paratype (CESS 318).

**Colour in ethanol**: Body uniform yellowish green to bright green; rostral, infralabials and the underside creamish white; creamish yellow to white ventral stripe along the notched ventral keels; slight discoloration in the preocular; the inter-scales are colored with a consecutive series of black and white stripes that slant towards the head; light blue coloration in the angle of the jaw and patches on the body (due to the preservative); yellow clouded with white eyes with black speckles; concentration of black speckles both in the anterior and posterior end of the dilated pupil; tongue yellowish (Fig 4A–4E).

**Maxillary arch dentition**: Maxillary bone mildly arched with a curve towards the anterior end of the prediastemal tooth set (Fig 2D); a total of 20 teeth that rise perpendicular to the maxilla and curve inward posteriorly; prediastemal teeth 13 and postdiastemal teeth 7, observable gradual tooth size increase in the prediastemal tooth set with a smaller tooth suffixing the largest tooth of the prediastemal set. A comparatively small diastema, 2–2.5 tooth sockets wide, suffixed with a set of 5 smaller teeth; a pair of large grooved teeth at the end.

**Hemipenal description**: Organ fully everted, examined in-situ. Organ short, thick, heavily flounced and ornamented with spines (Fig 4C). Pedicel slim, barely visible, 8.31 mm long, 6.41 mm wide, extending till 6^th^ subcaudal scale; lobe very wide and large, head not quite bilobed, flat; lobed head crown with small spines radiating towards sides; lobed body and sides with very large cursive spines, some spines half the size of the pedicel; sulcus spermaticus barely visible in sulcate view, being hidden by the protruding lobe spines.

**Variation shown by paratype**: Agreeing with the holotype is most respects, and exhibiting the following intraspecific variations: total length 1189 mm; snout to vent length 764 mm and tail length 425 mm; relative tail length 0.36; head length 22.4 mm; horizontal diameter of the eye 4.6 mm; vertical diameter of the eye 3.5 mm; distance from nostril to eye 5.9 mm; distance from snout tip to eye 8 mm; dorsal scale rows 13:13:13 (after neck, at midbody and before vent); ventrals 207; subcaudals 165, divided; temporals 3+3 (anterior + posterior) on the both side; three post-oculars (both left and right); supralabials 7–8, 4^th^ the largest, 6^th^ in contact with the eye; infralabials 9, 5^th^ and 6^th^ contacting posterior genials.

**Comparisons: *Proahaetulla* gen. nov**. differs from the genera in Ahaetuliinae with the combination of the following characters. *Dendrelaphis*–rostral lacking a protuberance; pupil circular; loreal region not strongly concave; ventral scales mildly notched; dorsum never completely verdant green; *Chrysopelea*–rostrum lacking a protuberance; pupil circular; loreal region not strongly concave; dorsum never verdant green; ventrals strongly notched; *Dryophiops*–dorsum never verdant green; dorsal scales smooth; posterior temporals not greater than two; loreals one on each side (Fig 1B–1F) (please refer key below).

Due to phenetic similarity and sympatric occurrence, the new genus is compared with the genus *Ahaetulla* at a species-level as follows: dorsal body scales distinctly keeled (vs. smooth in all *Ahaetulla* spp. except *A*. *perroteti*, that has faint keels on sacral rows); three postoculars (Figs 1B, 1C & 4E) on each side of head (vs. 2 in all *Ahaetulla* spp.); green dorsal colour (vs. always brown in *A*. *pulverulenta*, *A*. *fascioalata*, frequently brown in *A*. *fronticincta*, *A*. *anomala*); snout without rostral appendage (vs. with a rostral appendage in *A*. *nasuta*, *A*. *pulverulenta*, *A*. *anomala*); ventrals– 196–207 (vs. < 160 in *A*. *perroteti*, *A*. *dispar*); subcaudals– 160–165 (vs. < 120 in *A*. *dispar*, *A*. *perroteti*); loreals– 2 (vs. no loreal in *A*. *nasuta*, *A*. *perroteti*, *A*. *anomala*, *A*. *pulverulenta*, vs. 1–2 loreal(s) on each side of head in *A*. *dispar*, *A*. *prasina*) (S2 Table).

**Relationships**: The new lineage is a sister taxon to the genus *Ahaetulla*, as recovered in our phylogeny. Our tree topology varies slightly in comparison to previous reconstructions, likely due to the use of the different nucleotide substitution models for the analyses (Bayesian analysis with mixed substitution models vs. ML analysis with GTRG model). In addition, we were unable to include other available RAG1 sequences from the GenBank as they could not be aligned with our generated RAG1 sequences. Regardless of the variation in the topology within the *Ahaetulla* clade, there is very strong support for the node of interest (the relationship between *Proahaetulla* **gen. nov**. and *Ahaetulla*) in both the analyses. The generic status of *Proahaetulla* **gen. nov**. is further supported by the date of divergence (26.57 Ma) from its MRCA, indicating that it is the oldest lineage in the group, besides also differing from *Ahaetulla* and other genera in morphological characters.

**Fig 5:**
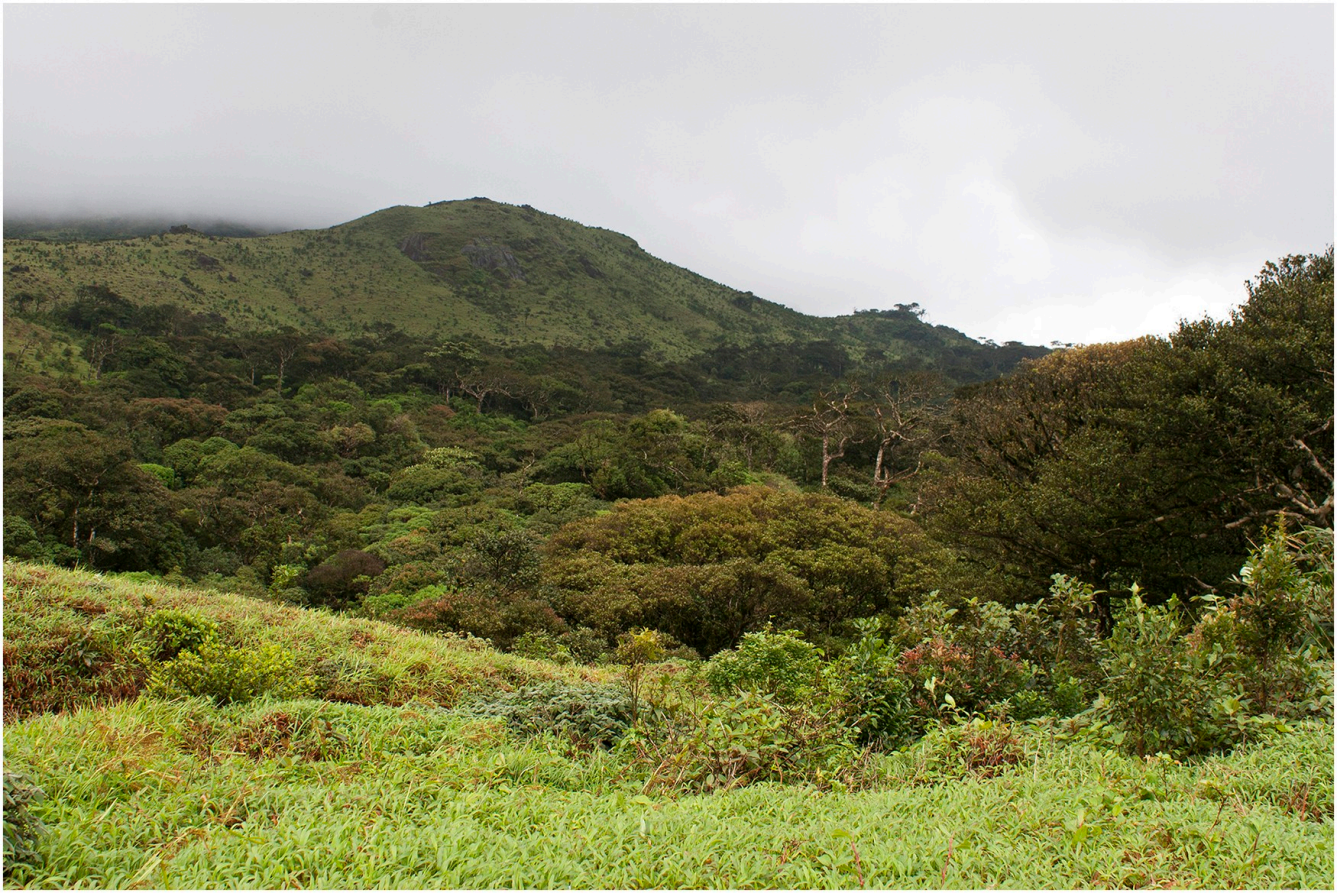
Habitat at the type locality of *Proahaetulla antiqua* gen. et sp. nov., showing montane rainforests atop Agasthyamalai hills, southern Western Ghats.

**Distribution**: The new taxon was found only in the far south of the Western Ghats. It occurs in Agasthyamalai hills, where it was recorded in the high elevation wet forests (> 1200 m asl) at Agasthyamalai peak and in Pandimotta (Fig 5). The new taxon’s range is likely to encompass other high elevation regions of Agasthyamalai. This taxon also broadly overlaps in its latitudinal distributional range with *A*. cf. *dispar* and *A*. cf. *nasuta*.

**Natural history**: The holotype was collected from a tree branch inside a forest patch at an elevation of 1640 msl, on the way to Agasthyar peak. It was found resting coiled on a tree at ca. 1640 h about 2.5 meters above the forest floor. The paratype was sighted at night around 2200 h, sleeping on a shrub at a height of about 2 m from the ground, inside a dense forest patch at an elevation of 1224 msl in Pandimotta ca. 25 km north of the type locality. Members of Ahaetuliinae are mostly arboreal snakes with a few outliers such as *Ahaetulla perroteti* and *A*.*dispar* with environmental adaptations and a body plan suited for a life in open montane grasslands. The body morphology of this genus is similar to arboreal *Ahaetulla* and *Dryophiops*. Since both the specimens were found inside thickly wooded forests resting and showing activity on trees and shrubs, we speculate that this taxon is also adapted to a completely arboreal lifestyle.

## Discussion

The Colubrid subfamily Ahaetuliinae is a diverse group of predominantly, arboreal snakes, distributed widely in the tropical regions of the Southeast Asia [21–23]. The species are currently grouped under four genera: *Ahaetulla* Link 1807, *Chrysopelea* Boie 1826, *Dendrelaphis* Boulenger, 1890 and *Dryophiops* Boulenger, 1896 [23]. The first three genera are widely distributed in Peninsular India, with high diversity in the Western Ghats region [17, 18, 25, 28]. In this work, we add a new endemic genus and species *Proahaetulla antiqua* **gen. et sp. nov**. for the Western Ghats.

Phylogenetically, the new genus was recovered, with strong nodal support, as sister to the genus *Ahaetulla*. The phylogenetic position and divergence dates reveal new insights into the historical biogeography of snakes of the subfamily Ahaetuliinae. Divergence dates show that the ancestors of *Proahaetulla* **gen. nov**. and *Ahaetulla* diverged during the Oligocene/ Early Miocene boundary and the discovery adds one of the oldest persisting monotypic lineages of snakes to the Western Ghats.

Distribution records suggest that the new lineage is potentially a narrow endemic occurring in the high elevations of the southernmost massif, the Agasthyamalai in the Western Ghats. Its geographic range roughly mirrors the restricted distributional range that many taxa have within the Agasthyamalai region [57, 58, 59]. For example, a deeply divergent monotypic lineage of agamid lizard *Microauris aurantolabium* is also known only from the high elevations of the Agasthyamalai [57] and a number of old lineages of frogs are also confined to Agasthyamalai [19]. The southern regions of the Western Ghats have long been recognized as rainforest refugia [60], though the exact extent of the refugia remains uncertain. However, local endemism in massifs such as Agasthyamalai suggests the possibility of multiple micro-refugia across the different massifs in the southern Western Ghats. This study provides further evidence that the Western Ghats served as a refugium in the past, including for the common ancestors of this clade comprising the genera *Ahaetulla* and *Proahaetulla*.

Our delimitation of this new lineage is in keeping with the recent consensus on the need for an integrative taxonomy, invoking multiple lines of evidence [60–63]. The present new lineage is distinct in its phylogenetic position, is deeply divergent (26.55 Ma, HPD 21.36–31.50) in being a sister lineage of all sampled *Ahaetulla*, and also differs in morphology from other genera in the same subfamily. Traditional methods of morphological analysis using PCA revealed large overlaps between the genera *Chrysopelea*, *Dendrelaphis*, *Ahaetulla*, *Dryophiops* making it difficult to separate any of these genera with quantitative external morphological characters. Considering the morphological synapomorphies and similarities between *Chrysopelea* and *Dendrelaphis*, it is not surprising that the new genus superficially resembles *Ahaetulla*.

With the addition of *Proahaetulla*, a number of unique patterns of character evolution can be highlighted (Table 3). The dorsal keeled scales that are shared among *Chrysopelea*, *Dendrelaphis*, *Dryophiops*, *and Proahaetulla* **gen. nov**. are lost in *Ahaetulla’s* ancestor. Pupil shape shows early evidence of divergence in Ahaetuliinae, with round pupils (*Chrysopelea*, *Dendrelaphis*) and horizontal pupils (*Ahaetulla*, *Dryophiops and Proahaetulla* **gen. nov**.). Apical pits are present in *Chrysopelea*, *Dendrelaphis*, *and Dryophiops*, but are lost in the common ancestor of *Ahaetulla* and *Proaheatulla*.

In addition, certain morphological characters shared by the clade composed of the three genera–*Dryophiops*, *Ahaetulla* and *Proahaetulla* **gen. nov**.–reveal interesting patterns of early divergence of characters during the ancestral split between this clade and its sister clade (*Chrysopelea*, *Dendrelaphis*) of Ahaetuliinae. The members of the clade, *Dryophiops*, *Ahaetulla* and *Proahaetulla* **gen. nov**., all possess a laterally compressed, elongated body plan, elongated sharp snout, large eyes with unique horizontal pupils paired with a well developed canthus rostralis for specialized binocular vision, which we hypothesize to have been acquired after the split between the two major clades of Ahaetuliinae.

We also highlight a unique convergence of character pattern divergence between two unrelated clades, Ahaetuliinae in Asia and the African clade comprising *Thelotornis*, *Dispholidus* and *Thrasops* occurring within Colubridae. As in the Asian clade, one of the genera in the African group (*Thelotornis*) has horizontal pupils (resembling *Ahaetulla*, *Proaheatulla* and *Dryophiops*), while the others (*Dispholidus* and *Thrasops*) have round pupils (resembling *Dendrelaphis* and *Chrysopelea*). In addition to these, there are several other genera referred to as ‘vine snakes’ across the world–*Thamnodynastes*, *Oxybelis*, *Philodryas*, *Xyelodontophis*, *Uromacer* and *Langaha*. It is noteworthy, that though unrelated, this group of snakes exhibits a vast array of uncanny morphological, behavioral and ecological convergences. These visual convergences led Boulenger to index these snake groups together in his catalogue of ophidians [64].

An additional finding from this study, the topology of our tree suggests that the genus *Ahaetulla* originated in Peninsular India. *Ahaetulla* is currently distributed along the Indian subcontinent and is found along South and Southeast Asia, including South China, Myanmar, Thailand Indonesia, Malaysia and western part of the Philippines island archipelago. The Genus *Dryophiops* occurs sympatrically with *Ahaetulla* throughout its range in SE Asia including Indonesia, Malaysia and the western part of the Philippines archipelago. Our study suggests the split of *Proahaetulla* **gen. nov**. with the rest of *Ahaetulla* in Peninsular India thus indicating the geographic origin of the genus *Ahaetulla* [24].

The Agasthyamalai hills have been previously surveyed for snakes, starting from the historical works of R.H. Beddome and Frank Wall in the 19^th^ and 20^th^ centuries. In recent times, there have been many surveys of snakes in Agasthyamalai [65–70] with new records (*Calliophis bibroni* and *C*. *beddomei* respectively) from this landscape [69–70]. Recent discoveries of a few new genera of arthropods [71–76], fishes [77–78], frogs [79], lizards [80–81] and birds [10] provide further evidence of the importance of the Western Ghats as a biodiversity hotspot. Our new finding once again underscores our limited knowledge about snake diversity and distribution patterns in the Western Ghats biodiversity hotspot.

*Proahaetulla* **gen. nov**. is the first deeply divergent colubrid snake genus reported in recent decades from the southern Western Ghats in the Indian peninsula. Despite new discoveries of frogs in recent years [59, 82], a new endemic genus of colubroid snake from the Western Ghats is a surprise, as the last such descriptions were at least a century ago, if not more–*Dieurostus* Berg, 1901; *Rhabdops* Boulenger, 1893 and *Xylophis* Beddome, 1878 –making *Proahaetulla* **gen. nov**. a once-in-a century find. Many new species of snakes have been described recently from Peninsular India across several genera (in *Rhabdops* [11]; Uropeltidae [15, 83–84]; *Xylophis* [14]; *Dendrelaphis* [17, 18]; *Lycodon* [85]; *Boiga* [86]; and *Calliophis* [16]). Several of these such as the new *Xylophis*, *Dendrelaphis*, *Boiga* and *Calliophis* are taxa which are long-known and previously sampled by researchers, but were misclassified or assigned to other similar genera. However, the present new taxon *Proahaetulla antiqua* **gen. et sp. nov**. is a completely new finding, which does not appear to have been previously encountered by the scientific community to the best of our knowledge.

### Key to Ahaetuliinae

1a) pupil horizontal; canthus rostralis strongly concave … 2a
1b) pupil rounded; canthus rostralis not strongly concave … 4a
2a) mid-dorsum with 13–15 rows of keeled scales …. *Proahaetulla* **gen. nov**. (1 species)
2b) mid-dorsum with not < 15 rows of smooth scales … 3a
3a) dorsal scales without apical pits; ventrals smooth … *Ahaetulla* (9 species)
3b) dorsal scales with apical pits; ventrals keeled … *Dryophiops* (2 species)
4a) mid-dorsum with 13–15 rows of scales; ventrals keeled … *Dendrelaphis* (45 species)
4b) mid-dorsum with 17 rows of keeled scales; ventrals notched … *Chrysopelea* (5 species)

## Supporting information

S1 Appendix Comparative material of Indian congeners examined. (DOCX)

S2 Appendix List of GenBank accession numbers for ingroup and outgroup taxa and locus used in this study. (DOCX)

S1 Table Details of gene regions amplified, PCR primers used, DNA sequences length (in base pairs) and references and protocol followed in this study. (DOCX)

S2 Table Comparison of morphological characters of *Ahaetulla* (Indian congeners) and other genera of Ahaetuliinae. (DOCX)

S3 Table List of external fossil records and secondary calibration used in this study to estimate the time of divergence of *Proahaetulla* gen. nov. and *Ahaetulla*. (DOCX)

S4 Table Details of PCA summary, loadings and scores. (DOCX)

## References

[1] Myers N, Mittermeier RA, Mittermeier CG, Da Fonseca GA, Kent J. Biodiversity hotspots for conservation priorities. Nature. 2000 2;403(6772):853–858. 10.1038/35002501 10706275

[2] Gunawardene NR, Daniels DA, Gunatilleke IAUN, Gunatilleke CVS, Karunakaran PV, Nayak GK, et al A brief overview of the Western Ghats–Sri Lanka biodiversity hotspot. Current Science. 2007 12;93(11):1567–1572.

[3] Murugan C, Manickam VS, Sundaresan V, Jothi GJ. *Miliusa tirunelvelica*, a new species of Annonaceae from the Kalakkad–Mundanthurai Tiger Reserve, Western Ghats, India. Novon. 2004 3;14:102–104.

[4] Narayanan MK, Ratheesh SM, Shareef T, Shaju AR, Sivu KA, Sujana MK, et al A new species of *Syzygium* (Myrtaceae) from the southern Western Ghats of Kerala, India. Int J Adv Res. 2014 3;2(3):1055–1058.

[5] Sivu AR, Aswathi P, Shaju T, Pradeep NS, Kumar ES. *Memecylon kurichiarensis* (Melastomataceae), a new species from the Western Ghats. Int J Adv Res. 2015 10;3(10):1086–1090.

[6] Page N, Nerlekar AN. A new species of *Miliusa* (Annonaceae) from the Western Ghats of Karnataka, India. Phytotaxa. 2016 1;245(1):79–83.

[7] Kumar AB, Raj S, Ng PKL. Description of a new genus and new species of a fully arboreal crab (Decapoda: Brachyura: Gecarcinucidae) from the Western Ghats, India, with notes on the ecology of arboreal crabs. J Crustac Biol. 2017 4;37(2):157–167.

[8] Sondhi Y, Kitching IJ, Basu DN, Kunte K. A new species of *Theretra* Hubner (Lepidoptera: Sphingidae) from the southern Western Ghats, India. Zootaxa. 2017 9;4323(2):185–196.

[9] Raghavan R, Tharian J, Ali A, Jadhav S, Dahanukar N. *Balitora jalpalli* a new species of stone loach (Teleostei: Cypriniformes: Balitoridae) from Silent Valley, southern Western Ghats, India. J Threat Taxa. 2013 3;5(5):3921–3934.

[10] Robin VV, Vishnudas CK, Gupta P, Rheindt FE, Hooper DM, Ramakrishnan U, et al Two new genera of songbirds represent endemic radiations from the Shola Sky Islands of the Western Ghats, India. BMC Evol Biol. 2017 1;17: 31 10.1186/s12862-017-0882-6 28114902 PMC5259981

[11] Giri VB, Deepak V, Captain A, Das A, Das S, Rajkumar KP, et al A new species of *Rhabdops* Boulenger, 1893 (Serpentes: Natricinae) from the northern Western Ghats region of India. Zootaxa. 2017 9;4319(1):27–52.

[12] Garg S, Biju SD. Description of four new species of Burrowing Frogs in the *Fejervarya rufescens* complex (Dicroglossidae) with notes on morphological affinities of *Fejervarya* species in the Western Ghats. Zootaxa. 2017 6;4277(4):451–490. 10.11646/zootaxa.4277.4.1 30308626

[13] Garg S, Suyesh R, Sandeep S, Biju SD. Seven new species of Night Frogs (Anura, Nyctibatrachidae) from the Western Ghats Biodiversity Hotspot of India, with remarkably high diversity of diminutive forms. Peer J. 2017 2;5:e3007 10.7717/peerj.3007 28243532 PMC5322763

[14] Gower DJ, Winkler JD. *Taxonomy* of the Indian snake *Xylophis* Beddome (Serpentes: Caenophidia), with description of a new species. Hamadryad. 2007 3;31(2):315–329.

[15] Gower DJ, Giri VB, Captain A, Wilkinson M. A reassessment of *Melanophidium* Günther, 1864 (Squamata: Serpentes: Uropeltidae) from the Western Ghats of peninsular India, with the description of a new species. Zootaxa. 2016 3;4085:481–503. 10.11646/zootaxa.4085.4.2 27394315

[16] Smith EN, Ogale H, Deepak V, Giri VB. A new species of coralsnake of the genus *Calliophis* (Squamata: Elapidae) from the west coast of peninsular India. Zootaxa. 2012 3;3437:51–68.

[17] Vogel G, van Rooijen J. Contributions to a Review of the *Dendrelaphis pictus* (Gmelin, 1789) Complex (Serpentes: Colubridae)—3. The Indian Forms, with the Description of a New Species from the Western Ghats. J Herpetol. 2011a 3;45(1):100–110.

[18] Vogel G, van Rooijen J. A new species of *Dendrelaphis* (Serpentes: Colubridae) from the Western Ghats—India. Taprobanica. 2011b 10;3(02):77–85.

[19] Vijayakumar SP, Menezes RC, Jayarajan A, Shanker K. Glaciations, gradients and geography: multiple drivers of diversification of bush frogs in the Western Ghats Escarpment. Proc. R. Soc. B. 2016;282:20161011. 10.1098/rspb.2016.1011 PMC5013767 27534957

[20] Srikanthan AN, Swamy P, Mohan AV, Pal S. A distinct new species of riparian rock-dwelling gecko (genus: *Hemidactylus*) from the southern Western Ghats. Zootaxa. 2018 6;4434(1):141–157. 10.11646/zootaxa.4434.1.9 30313205

[21] Pyron RA, Burbrink FT, Wiens JJ. 2013. A phylogeny and revised classification of Squamata, including 4161 species of lizards and snakes. BMC Evol Biol. 2013 4;13:93 10.1186/1471-2148-13-93 23627680 PMC3682911

[22] Zheng Y, Wiens JJ. Combining phylogenomic and supermatrix approaches, and a time-calibrated phylogeny for squamate reptiles (lizards and snakes) based on 52 genes and 4162 species. Mol Phylogenet Evol. 2016 1; 94: 537–547. 10.1016/j.ympev.2015.10.009 26475614

[23] Figueroa A, McKelvy AD, Grismer LL, Bell CD, Lailvaux SP. A Species-Level Phylogeny of Extant Snakes with Description of a New Colubrid Subfamily and Genus. PLoS ONE. 2016 9;11(9):e0161070 10.1371/journal.pone.0161070 27603205 PMC5014348

[24] Mallik AK. Systematics and comparative biogeography of vine snakes (Genus: Ahaetulla, Family: Colubridae) and pit vipers (Genus: Trimeresurus, Family: Viperidae) in Peninsular India. Ph.D. thesis. Indian Institute of Science, Bangalore. 2018.

[25] Whitaker R, Captain A. Snakes of India–the field guide. India: Chengalpattu; Draco Books 2004.

[26] Miralles A, David P. First record of *Ahaetulla mycterizans* (Linnaeus, 1758) (Reptilia, Squamata, Colubridae) from Sumatra, Indonesia, with an expanded definition. Zoosystema. 2010 9;32(3):449–456.

[27] Mohapatra PP, Dutta SK, Kar NB, Das A, Murthy BH, Deepak V. *Ahaetulla nasuta anomala* (Annandale, 1906)(Squamata: Colubridae), resurrected as a valid species with marked sexual dichromatism. Zootaxa. 2017 5;4263(2):318–332. 10.11646/zootaxa.4263.2.6 28609871

[28] Smith MA. The Fauna of British India, Ceylon and Burma. Reptilia and Amphibia Vol. 3 Serpentes. London: Taylor & Francis; 1943.

[29] Townsend T, Larson A, Louis E, Macey JR. Molecular Phylogenetics of Squamata: The Position of Snakes, Amphisbaenians, and Dibamids, and the Root of the Squamate Tree. Systematic Biology. 2004 10;53(5):735–57. 10.1080/10635150490522340 15545252

[30] Wüster W, Crookes S, Ineich I, Mané Y, Pook CE, Trape J, et al The phylogeny of cobras inferred from mitochondrial DNA sequences: Evolution of venom spitting and the phylogeography of the African spitting cobras (Serpentes: Elapidae: *Naja nigricollis* complex). Molecular Phylogenetics and Evolution. 2007 8;45:437–453. 10.1016/j.ympev.2007.07.021 17870616

[31] Parkinson CL, Moody SM, Alquist JE. Phylogenetic relationships of the ‘Agkistrodon Complex’ based on mitochondrial DNA sequence data. Pp. 63–78. In Thorpe RS, Wüster W, Malhotra A (Eds.). Venomous Snakes: Ecology, Evolution, and Snakebite Symposia of the Zoological Society of London. U.K. Oxford, Clarendon Press. 1997:63–78.

[32] Dubey B, Meganathan PR, Vidal N, Ikramul H. Molecular Evidence for the Nonmonophyly of the Asian Natricid Genus *Xenochrophis* (Serpentes, Colubroidea) as Inferred from Mitochondrial and Nuclear Genes. Journal of Herpetology. 2012;46(2):263–268.

[33] Tamura K, Peterson D, Peterson N, Stecher G, Nei M, Kumar S. MEGA5: Molecular evolutionary genetics analysis using maximum likelihood, evolutionary distance, and maximum parsimony methods. Mol Biol Evol. 2012 5;28(1071):2731–2739. 10.1093/molbev/msr121 PMC3203626 21546353

[34] Edgar RC. MUSCLE: multiple sequence alignment with high accuracy and high throughput. Nucleic Acids Res. 2004 2;32:1792–1797. 10.1093/nar/gkh340 15034147 PMC390337

[35] Parkinson CL. Molecular systematics and biogeographical history of the pitvipers as determined by mitochondrial ribosomal sequences. Copeia. 1999 8;(3):576–586.

[36] Kjer KM. Use of rRNA secondary structure in phylogenetic studies to identify homologous positions: an example of alignment and data presentation from the frogs. Mol. Phylogenet. Evol. 1995 10;4:314–330. 10.1006/mpev.1995.1028 8845967

[37] Gutell RR. Collection of small subunit (16S- and 16S-like) ribosomal RNA structures. Nucleic Acids Res. 1994 10;22:3502–3507. 10.1093/nar/22.17.3502 7524024 PMC308311

[38] Lanfear R, Calcott B, Ho SYW, Guindon S. PartitionFinder: combined selection of partitioning schemes and substitution models for phylogenetic analyses. Mol Biol Evol. 2012 1;29(6):1695–1701. 10.1093/molbev/mss020 22319168

[39] Silvestro D, Michalak I. raxmlGUI: a graphical front-end for RAxML. Org Div Evol. 2012 12;12(4):335–337.

[40] Stamatakis A. RAxML-VI-HPC: maximum likelihood-based phylogenetic analyses with thousands of taxa and mixed models. Bioinformatics. 2006 8;22:2688–2690. 10.1093/bioinformatics/btl446 16928733

[41] Ronquist F, Teslenko M, van der Mark P, Ayres DL, Darling A, Höhna S, et al MrBayes 3.2: Efficient Bayesian Phylogenetic Inference and Model Choice Across a Large Model Space. Syst Biol. 2012 2;61(3):539–542. 10.1093/sysbio/sys029 22357727 PMC3329765

[42] Miller MA, Pfeiffer W, Schwartz T. "Creating the CIPRES Science Gateway for inference of large phylogenetic trees" in Proceedings of the Gateway Computing Environments Workshop (GCE). LA: New Orleans. 2010.

[43] Rambaut A, Suchard MA, Xie D, Drummond AJ. Tracer v1.6, http://tree.bio.ed.ac.uk/software/tracer/. 2016.

[44] Rambaut A. FigTree v. 1.4. http://tree.bio.ed.ac.uk/software/figtree. 2012.

[45] Drummond AJ, Rambaut A. Beast: Bayesian evolutionary analysis by sampling trees. BMC Evol Biol. 2007 11;7:214 10.1186/1471-2148-7-214 17996036 PMC2247476

[46] Drummond AJ, Suchard MA, Xie D, Rambaut A. Bayesian phylogenetics with BEAUti and the BEAST 1.7. Mol Biol Evol. 2012 2;29(8):1969–1973. 10.1093/molbev/mss075 22367748 PMC3408070

[47] Gernhard T. The conditioned reconstructed process. J Theoret Biol. 2008 8;253:769–778. 18538793 10.1016/j.jtbi.2008.04.005

[48] Drummond AJ, Ho SYW, Phillips MJ, Rambaut A. Relaxed phylogenetics and dating with confidence. PLoS Biol. 2006 3;4(5):699–710. 10.1371/journal.pbio.0040088 PMC1395354 16683862

[49] Alencar LRV, Quental TB, Grazziotin FG, Alfaro ML, Martins M, Venzon M, et al Diversification in vipers: Phylogenetic relationships, time of divergence and shifts in speciation rates. Mol Phylogenet Evol. 2016 8;105:50–62. 10.1016/j.ympev.2016.07.029 27480810

[50] Holman JA. Fossil Snakes of North America: Origin, Evolution, Distribution, Paleoecology. Bloomington: Indiana University Press 2000.

[51] Pyron RA, Burbrink FT. Extinction, ecological opportunity, and the origins of global snake diversity. Evolution. 2012 1;66:163–178. 10.1111/j.1558-5646.2011.01437.x 22220872

[52] Chen X, Huang S, Guo P, Colli GR, Nieto Montes de Oca A, Vitt LJ, et al Understanding the formation of ancient intertropical disjunct distributions using Asian and Neotropical hinged-teeth snakes (*Sibynophis* and *Scaphiodontophis*: Serpentes: Colubridae). Mol Phylogenet Evol. 2013 1; 66(1):254–261. 10.1016/j.ympev.2012.09.032 23044403

[53] Burbrink FT, Lawson R. How and when did Old World ratsnakes disperse into the New World? Mol Phylogenet Evol. 2007 9;43(1):173–189. 10.1016/j.ympev.2006.09.009 17113316

[54] Rage JC, Folie A, Rana RS, Singh H, Rose KD, Smith T. A diverse snake fauna from the early Eocene of Vastan Lignite Mine, Gujarat, India. Acta Palaentologica Polonica. 2008 9;53(3):391–403.

[55] Dowling HG. A proposed standard system of counting ventrals in snakes. Brit J Herpetol. 1951;1:97–99.

[56] Hammer Ø, Harper DAT, Ryan PD. PAST: Paleontological Statistics Software Package for Education and Data Analysis. Palaeontologia Electronica. 2001 6;4(1):9.

[57] Pal S, Vijayakumar SP, Shanker K, Jayarajan A, Deepak V. A systematic revision of *Calotes* Cuvier, 1817 (Squamata: Agamidae) from the Western Ghats adds two genera and reveals two new species. Zootaxa. 2018;4482(3):401–450. 10.11646/zootaxa.4482.3.1 30313808

[58] Das I. A Photographic Guide to snakes and other reptiles of India. UK Ltd: New Holland Publisher 2002;144 pp.

[59] Abraham RK, Pyron RA, Ansil BR, Zachariah A, Zachariah A. Two novel genera and one new species of treefrog (Anura: Rhacophoridae) highlight cryptic diversity in the Western Ghats of India. Zootaxa. 2013 2;3640(2):177–189. 26000411 10.11646/zootaxa.3640.2.3

[60] Prasad V, Farooqui A, Tripathi SM, Garg R, Thakur B. Evidence of late Palaeocene-early Eocene equatorial rain forest refugia in southern Western Ghats, India. Journal of Biosciences. 2009;34(5):777 20009271 10.1007/s12038-009-0062-y

[61] Padial JM, Miralles A, De la Riva I, Vences M. The integrative future of taxonomy. Frontiers in zoology. 2010;7(1):16. 20500846 10.1186/1742-9994-7-16 PMC2890416

[62] Schlick-Steiner BC, Steiner FM, Seifert B, Stauffer C, Christian E, Crozier RH. Integrative taxonomy: a multisource approach to exploring biodiversity. Annual Review of Entomology. 2010 1;55:421–38. 10.1146/annurev-ento-112408-085432 19737081

[63] Shanker K, Vijayakumar SP, Ganeshaiah KN. Unpacking the species conundrum: philosophy, practice and a way forward. Journal of genetics. 2017 7;96(3):413–30. 28761006 10.1007/s12041-017-0800-0

[64] Boulenger GA. Catalogue of the Snakes in the British Museum, (Natural History): Colubridæ (Opisthoglyphæ and Proteroglyphæ), Amblycephalidæ, and Viperidæ. London 1893;3.

[65] Inger RF, Shaffer HB, Koshy M, Bakde KR. A report on a collection of amphibians and reptiles from the Ponmudi, Kerala, South India. J Bombay Nat Hist Soc. 1984;81(2):406–427 & 551–570.

[66] Cherian PT, Rema D, Ravichandran S. Ichthyo and Herpetofaunal diversity of Kalakkad Wildlife Sanctuary. Zoo’s Print J. 2000 2;15(2):203–206.

[67] Ishwar NM, Chellam R, Kumar A. Distribution of forest floor reptiles in the rainforest of Kalakkad-Mundanthurai Tiger Reserve, South India. Curr Sci. 2001 2;80(3):413–418.

[68] Chandramouli SR, Ganesh SR. Herpetofauna of Southern Western Ghats, India–reinvestigated after decades. Taprobanica. 2010 3;2(2):8–21.

[69] Jins VJ, Bhupathy S, Panigrahi M. New record of Beddome’s Coral Snake, *Calliophis beddomei* Smith 1943 from the southern Western Ghats, India. Herpetology Notes 7, 2014 10;7:555–557.

[70] Deepak V, Harikrishnan S, Vasudevan K, Smith EN. Redescription of Bibron’s Coralsnake, *Calliophis bibroni* Jan 1858 with notes and new records from south of the Palghat and Shencottah gaps of the Western Ghats, India. Hamadryad. 2010 11;35(1):1–10.

[71] Patwardhan A, Schimmel R, Athalye RP. A New Genus *Punctodensus* from North Western Ghat, Maharashtra, India (Coleoptera: Elateridae: Agrypninae: Hemirrhipini). Genus. 2009;20(1):53–60.

[72] Siliwal M, Gupta N, Raven R. CEPF Western Ghats Special Series: A new genus of the family Theraphosidae (Araneae: Mygalomorphae) with description of three new species from the Western Ghats of Karnataka, India. Journal of Threatened Taxa. 2012;14(4):3233–3254.

[73] Mirza ZA, Sanap RV, Bhosale H. Preliminary Review of Indian Eumenophorinae (Araneae: Theraphosidae) with Description of a New Genus and Five New Species from the Western Ghats. PLOS ONE. 2014;9(2):e87928 10.1371/journal.pone.0087928 24551072 PMC3925112

[74] Singh S, Devi OK, Srinivasa YB. Description of a new genus and three species of Encyrtidae (Hymenoptera: Chalcidoidea) from the Western Ghats of Karnataka, India. Zootaxa. 2014 6;3(3814):369–384. 10.11646/zootaxa.3814.3.4 24943434

[75] Pati SK, Sharma RM. Description of Ghatiana, a new genus of freshwater crab, with two new species and a new species of *Gubernatoriana* (Crustacea: Decapoda: Brachyura: Gecarcinucidae) from the Western Ghat Mountains, India. Journal of Natural History. 2014 2; 48(21–22):1279–1298.

[76] Selvakumar C, Sivaruban T, Subramanian KA, Sivaramakrishnan KG. A new genus and species of Atalophlebiinae (Insecta: Ephemeroptera: Leptophlebiidae) from Palni hills of the southern Western Ghats, India. Zootaxa. 2016 11;4208(4):381–391. 10.11646/zootaxa.4208.4.5 28006814

[77] Raghavan R, Philip S, Ali A, Dahanukar N. *Sahyadria*, a new genus of barbs (Teleostei: Cyprinidae) from Western Ghats of India. Journal of Threatened Taxa. 2013 11;5(15):4932–4938.

[78] Pramod PK, Fang F, Devi KR, Liao T, Indra TJ, Beevi KSJ, et al *Betadevario Ramachandrani*, A New Danionine Genus and species from the Western Ghats of India (Teleostei: Cyprinidae: Danioninae). Zootaxa. 2010 6; 2519(1):31–47.

[79] Biju SD, Van Bocxlaer I, Giri VB, Simon L, Bossuyt F. Two new endemic genera and a new species of toad (Anura: Bufonidae) from the Western Ghats of India. BMC Research Notes. 2009 12;2(241):1–6. 19968866 10.1186/1756-0500-2-241 PMC2797014

[80] Deepak V, Giri VB, Kazi MA, Dutta SK, Vyas R, Zambre AM, et al Systematics and phylogeny of *Sitana* (Reptilia: Agamidae) of Peninsular India, with the description of one new genus and five new species. Contributions to Zoology. 2016;85(1):67–111.

[81] Eremchenko VK, Das I. *Kaestlea*: a new genus of scincid lizards (Scincidae: Lygosominae) from the Western Ghats, south-western India. Hamadryad. 2004;28(1&2):43–50.

[82] Vijayakumar SP, Pyron RA, Dinesh KP, Torsekar VR, Achyuthan NS, Swamy P, et al A new ancient lineage of frog (Anura: Nyctibatrachidae: Astrobatrachinae subfam. nov.) endemic to the Western Ghats of Peninsular India. PeerJ 7:e6457 10.7717/peerj.6457 30881763 PMC6419720

[83] Aengals R, Ganesh SR. *Rhinophis goweri*—A New Species of Shieldtail Snake from the Southern Eastern Ghats, India. Russ J Herpetol. 2013 3;20(1):61–65.

[84] Jins VJ, Sampaio FL, Gower DJ. A New Species of *Uropeltis* Cuvier, 1829 (Serpentes: Uropeltidae) from the Anaikatty Hills of the Western Ghats of India. Zootaxa. 2018 5;4415(3);401–422. 10.11646/zootaxa.4415.3.1 30313609

[85] Mukherjee D, Bhupathy S. A new species of wolf snake (Serpentes: Colubridae: *Lycodon*) from Anaikatti Hills, Western Ghats, Tamil Nadu, India. Russian J Herpetol. 2007 4;14(1):21–26.

[86] Vogel G, Ganesh SR. A New Species of Cat Snake (Reptilia: Serpentes: Colubridae: *Boiga*) from Dry Forests of eastern Peninsular India. Zootaxa. 2013 4;3637(2):158–168. 26046190 10.11646/zootaxa.3637.2.6

