# Supplementary material for: Discovery of a deeply divergent new lineage of vine snake (Colubridae: Ahaetuliinae: Proahaetulla gen. nov.) from the southern Western Ghats of Peninsular India with a revised key for Ahaetuliinae
Source: PLoS One. 2019 Jul 17;14(7):e0218851. doi: 10.1371/journal.pone.0218851 (PMC6636718; doi:10.1371/journal.pone.0218851)
Supplement: S1 Appendix — (DOCX) [file pone.0218851.s001.docx]

**S1 Appendix. Comparative material of Indian congeners examined:**

*Ahaetulla* cf. *nasuta*

CESS 329; adult male; Kakki, Ranni Forest Division, Kerala; *Coll.* S.R. Chandramouli, 2012.

CESS 404; adult female; Idukki Wild Life Sanctuary, Kerala; *Coll.* S.R. Chandramouli, 2012.

CESS 089; adult female; Kurchermala, Kalpetta, Kerala; *Coll.* Ashok Kumar Mallik, 2010.

CESS 072; adult female; Harubi, Kudhremukh National Park, Karnataka; *Coll.* Ashok Kumar Mallik, 2010.

CESS 059; adult female; Amboli, Maharashtra; *Coll.* Ashok Kumar Mallik, 2009.

CESS 104; adult female; Matheran, Maharashtra; *Coll.* Ashok Kumar Mallik, 2010.

*Ahaetulla dispar*

CESS 187; adult female; CESS 188; adult male; CESS 189 adult male; Anaimudi shola National Park, Kerala; *Coll.* Ashok Kumar Mallik, 2011.

CESS 261; adult female; Achankovil/ Devarmala, Kerala; *Coll.* S.P Pal, 2011.

*Ahaetulla* cf. *pulverulenta*

CESS 159; adult female; Bhagavathi, Kudremukh National Park, Karnataka; *Coll.* Ashok Kumar Mallik, 2010.

*Ahaetulla perroteti*

CESS 286; adult female; Upper Bhavani, Mukurthi National Park, Tamilnadu; *Coll.* Ashok Kumar Mallik, 2011.

*Ahaetulla prasina*

CESS 340; adult male; Kane Wildlife Sanctuary, Arunachal Pradesh; *Coll.* Ashok Kumar Mallik, 2012.

CESS 347; adult male; Kane Wildlife Sanctuary, Arunachal Pradesh; *Coll.* Ashok Kumar Mallik, 2012.
