# Supplementary material for: Discovery of a deeply divergent new lineage of vine snake (Colubridae: Ahaetuliinae: Proahaetulla gen. nov.) from the southern Western Ghats of Peninsular India with a revised key for Ahaetuliinae
Source: PLoS One. 2019 Jul 17;14(7):e0218851. doi: 10.1371/journal.pone.0218851 (PMC6636718; doi:10.1371/journal.pone.0218851)
Supplement: S2 Appendix — (DOCX) [file pone.0218851.s002.docx]

**S2 Appendix. List of GenBank accession numbers for ingroup and outgroup taxa and locus used in this study**.

| **Tentative species identity** | **Cytb** | **16S** | **ND4** | **C-*mos*** | **RAG1** |
| --- | --- | --- | --- | --- | --- |
| *Proahaetulla antiqua* ***gen. et sp. nov.*** (CESS 259) | MH779628 | MH779632 | MH779629 | MH779630 | MH779631 |
| *Proahaetulla antiqua* ***gen. et sp. nov.*** (CESS 318) | MH779633 | MH779636 | MH779634 | MH779635 |  |
| *Ahaetulla nasuta* | KC347453 |  | KC347526 | KC347377 | KC347415 |
| *Ahaetulla pulverulenta* | KC347454 | KC347339 | KC347512 | KC347378 | KC347416 |
| *Ahaetulla fronticincta* | KX660447 | KX660173 | KX660576 | KX660312 |  |
| *Ahaetulla nasuta* | KX660467 | KX660194 | KX660594 | KX660333 | KX660096 |
| *Ahaetulla fasciolata* | KX660477 | KX660203 | KX660602 | KX660343 | KX660103 |
| *Ahaetulla mycterizans* | KX660479 | KX660205 | KX660604 | KX660345 | KX660105 |
| *Ahaetulla prasina* | KX660480 | KX660206 | KX660605 | KX660346 | KX660106 |
| *Ahaetulla prasina* | LC105637 |  |  |  |  |
| *Ahaetulla prasina* | KC010339 |  |  | KC010300 |  |
| *Ahaetulla prasina* | KC010338 |  |  | KC010299 |  |
| *Ahaetulla prasina* |  | KX660195 | KX660595 | KX660334 | KX660097 |
| *Ahaetulla prasina* |  | KX694615 |  | KX694801 |  |
| *Ahaetulla anomala* | KY769196 | KY769197 |  |  | KY769199 |
| *Dryophiops rubescens* | KX660526 | KX660256 |  | KX660397 |  |
| *Dryophiops philippina* | KX660517 | KX660247 | KX660641 | KX660388 | KX660139 |
| *Chrysopelea taprobanica* | KC347459 | KC347354 | KC347508 | KC347394 | KC347432 |
| *Chrysopelea ornata* | KC347481 | KC347353 | KC347496 | KC347393 | KC347431 |
| *Chrysopelea paradisi* | GQ895858 |  |  | GQ895802 |  |
| *Chrysopelea pelias* | KX660490 | KX660218 | KX660616 | KX660359 |  |
| *Dendrelaphis schokari* | KC347461 | KC347358 | KC347497 | KC347397 | KC347435 |
| *Dendrelaphis tristis* | KC347462 | KC347359 | KC347493 | KC347398 | KC347436 |
| *Dendrelaphis caudolineatus* | GQ895864 |  |  | GQ895808 |  |
| *Dendrelaphis subocularis* | KX660494 | KX660225 | KX660623 | KX660366 |  |
| *Dendrelaphis cyanochloris* | KX660454 | KX660181 | KX660580 | KX660320 |  |
| *Dendrelaphis marenae* | KX660514 | KX660244 | KX660640 | KX660385 |  |
| *Dendrelaphis fuliginosus* | KX660512 | KX660242 | KX660639 | KX660383 |  |
| *Dendrelaphis striatus* | KX660495 | KX660226 | KX660624 | KX660367 |  |
| *Dendrelaphis haasi* | KX660493 | KX660224 | KX660622 | KX660365 |  |
| *Dendrelaphis formosus* | KX660472 | KX660199 | KX660598 | KX660338 |  |
| *Dendrelaphis pictus* | KX660455 | KX660182 | KX660581 | KX660321 |  |
| *Dendrelaphis ngansonensis* |  | KX660158 | KX660561 | KX660297 |  |
| **OUTGROUP** | | | | | |
| *Hemorrhois hippocrepis* | AY486916 | AY643350 | AY487045 | AY486940 |  |
| *Hemorrhois algirus* | AY486911 | AY643349 | AY487037 | AY486935 |  |
| *Hemorrhois nummifer* | AY376742 | AY376771 | AY487049 | AY376800 |  |
| *Eirenis modestus* | AY486933 | AY376780 | AY487072 | AY486957 |  |
| *Dolichophis caspius* | HM210787 | AY376768 | AY487039 | AY376797 |  |
| *Zamenis lineatus* | HQ392567 |  | DQ902319 | DQ902099 |  |
| *Zamenis situla* | DQ902125 |  | DQ902303 | DQ902083 |  |
| *Zamenis persicus* | DQ902117 |  | DQ902297 | DQ902075 |  |
| *Lycodon osmanhilli* |  | KC347364 | KC347524 | KC347403 | KC347441 |
| *Oligodon arnensis* | KC347464 | KC347365 | KC347504 | KC347404 | KC347442 |
| *Xenochrophis piscator* | GQ225659 |  |  | GQ225669 | EU402868 |
| *Rhabdophis subminiatus* | GQ281777 | AF544805 | JQ687411 | JQ687436 |  |
| *Natrix natrix* | AY866537 | AF158530 | AY873736 | AF471121 | EU402858 |
| *Opisthotropis cheni* | GQ281779 |  | JQ687416 | JQ687441 |  |
| *Naja naja* | EU624299 | EU624270 | AY713378 | GQ225673 |  |
| *Naja kaouthia* | EU624298 | EU624269 | EU624209 | AY058938 | EU402857 |
| *Naja nigricollis* | EU624300 | EU624271 | AY713377 |  |  |
| *Ophiophagus hannah* | AF217842 | Z46480 | AY058984 | AY058940 |  |
| *Lycophidion capense* | AY612075 | AY611893 | FJ404376 | FJ404279 | EU402855 |
| *Duberria lutrix* | KX694892 | KX694640 | DQ486308 | DQ486161 |  |
| *Leioheterodon madagascariensis* | KX694881 | AY188061 | FJ404371 | KX694824 | AY487377 |
| *Psammophis mossambicus* | KX694878 | KX694660 | DQ486283 | DQ486185 |  |
| *Xenodermus javanicus* | AY425810 | AF544810 | U49320 | AF544711 | EU402869 |
| *Sistrurus catenatus* | AY223610 | AF057274 | AY223648 | KF410311 |  |
| *Sistrurus miliarius* | AY223611 | AF057275 | U41889 |  |  |
| *Crotalus durissus* | EU624302 | EU624274 | AY704885 |  |  |
| *Crotalus tigris* | AY223606 | AF057270 | AF156574 | JN620900 | JN621007 |
| *Bothrops diporus* | DQ305472 | DQ305454 | DQ305489 |  |  |
| *Daboia russellii* | DQ305459 | DQ305436 | DQ305477 |  |  |
| *Echis carinata* | GQ359433 | GQ359682 | GQ359521 |  |  |
