# Supplementary material for: Discovery of a deeply divergent new lineage of vine snake (Colubridae: Ahaetuliinae: Proahaetulla gen. nov.) from the southern Western Ghats of Peninsular India with a revised key for Ahaetuliinae
Source: PLoS One. 2019 Jul 17;14(7):e0218851. doi: 10.1371/journal.pone.0218851 (PMC6636718; doi:10.1371/journal.pone.0218851)
Supplement: S1 Table — (DOCX) [file pone.0218851.s003.docx]

**S1 Table. Details of gene regions amplified, PCR primers used, length DNA sequences (in base pairs) and protocol followed in this study.**

| **Gene region** | **Primer** | **Sequence 5'-3'** | **Seq. length** | **References** |
| --- | --- | --- | --- | --- |
| Cytb | Gludg  H16064 | TGACTTGAARAACCAYCGTTG  CTTTGGTTTACAAGAACAATGCTTTA | 1048bp | [1]  [2] |
| 16S rRNA | L2510  H3059 | CGCCTGTTTATCAAAAACAT  CCGGTCTGAACTCAGATCACGT | 472bp | [1] |
| ND4 | ND4  Leu | CACCTATGACTACCAAAAGCTCATGTAGAAGC  CATTACTTTTACTTGGATTTGCACCA | 663bp | [3] |
| C-*mos* | S77  S78 | CATGGACTGGGATCAGTTATG  CCTTGGGTGTGATTTTCTCACCT | 552bp | [4] |
| RAG1 | RAG1_f1a  RAG1_r2 | CAGCTGYAGCCARTACCATAAAAT  CTTTCTAGCAAAATTTCCATTCAT | 855bp | [5] |

References

1. Palumbi SR. Nucleic acids II: the polymerase chain reaction. In: Hillis, D.M., Moritz, C., Mable, B.K. (Eds.). Molecular Systematics. USA: Massachusetts, Sunderland; Sinauer Associates, Inc. 1996.
2. Burbrink FT, Lawson R, Slowinski JB. Mitochondrial DNA phylogeography of the polytypic north American rat snake (Elaphe obsoleta): a critique of the subspecies concept. Evolution. 2000;54:2107–2118.
3. Arévalo E, Davis SK, Sites JW. Mitochondrial DNA sequence divergence and phylogenetic relationships among eight chromosome races of the Sceloporus grammicus complex (Phrynosomatidae) in Central Mexico. Syst. Biol. 1994;43:387–418.
4. Lawson R, Slowinski JB, Crother BI, Burbrink FT. Phylogeny of the Colubroidea (Serpentes): New evidence from mitochondrial and nuclear genes, Mol Phylo et Evol. 2005; 37(2):581-601.
5. Wien JJ, Caitlin A. Kuczynsk, Smith SA, Mulcahy DG, Sites Jr JW, Townsend TM, Reeder TW. Branch Lengths, Support, and Congruence: Testing the Phylogenomic Approach with 20 Nuclear Loci in Snakes. Systematic Biology. 2008; 57 (3):420-431.
