# Supplementary material for: Discovery of a deeply divergent new lineage of vine snake (Colubridae: Ahaetuliinae: Proahaetulla gen. nov.) from the southern Western Ghats of Peninsular India with a revised key for Ahaetuliinae
Source: PLoS One. 2019 Jul 17;14(7):e0218851. doi: 10.1371/journal.pone.0218851 (PMC6636718; doi:10.1371/journal.pone.0218851)
Supplement: S2 Table — (DOCX) [file pone.0218851.s004.docx]

**S2 Table. Comparison of morphological characters of *Ahaetulla* (Indian congeners) and other genera of Ahaetuliinae.**

| **Species** | **Voucher no.** | **V** | **SC** | **SL^@^** | **SL2** | **L** | **PRSO** | **IL** | **PO** | **M** | **Keels** | **Max. teeth** |
| --- | --- | --- | --- | --- | --- | --- | --- | --- | --- | --- | --- | --- |
| *Proahaetulla antiqua* **gen. et sp. nov.** | CESS259 | 196 | 160 | 14 | 4 | 4 | 0 | 16 | 5 | 15 | 12 | 20 |
| *Proahaetulla antiqua* **gen. et sp. nov.** | CESS318 | 207 | 165 | 15 | 4 | 4 | 0 | 18 | 6 | 13 | 12 |  |
| *Ahaetulla dispar* | CESS187 | 141 | 103 | 16 | 2 | 0 | 4 | 18 | 4 | 15 | 0 | 12-16 |
| *Ahaetulla dispar* | CESS188 | 147 | 113 | 16 | 2 | 0 | 4 | 18 | 4 | 15 | 0 |  |
| *Ahaetulla dispar* | CESS189 | 153 | 110 | 16 | 2 | 2 | 4 | 18 | 4 | 15 | 0 |  |
| *Ahaetulla dispar* | CESS261 | 155 | 125 | 16 | 2 | 2 | 4 | 18 | 4 | 15 | 0 |  |
| *A.*cf. *nasuta* | CESS329 | 178 | 159 | 16 | 2 | 0 | 4 | 18 | 4 | 15 | 0 |  |
| *A.*cf. *nasuta* | CESS404 | 167 | 135 | 16 | 2 | 0 | 4 | 18 | 4 | 15 | 0 |  |
| *A.*cf*. nasuta* | CESS089 | 168 | 124 | 16 | 2 | 0 | 4 | 18 | 4 | 15 | 0 |  |
| *A.*cf. *nasuta* | CESS072 | 177 | 142 | 16 | 2 | 0 | 4 | 20 | 4 | 15 | 0 |  |
| *A.*cf. *nasuta* | CESS059 | 178 | 151 | 16 | 2 | 0 | 4 | 16 | 4 | 15 | 0 |  |
| *A.*cf. *nasuta* | CESS104 | 181 | 142 | 16 | 2 | 0 | 4 | 18 | 4 | 15 | 0 |  |
| *A.* cf. *pulverulenta* | CESS159 | 188 | 189 | 16 | 2 | 0 | 4 | 16 | 4 | 15 | 0 |  |
| *A. perroteti* | CESS286 | 140 | 84 | 16 | 4 | 0 | 0 | 18 | 2 | 15 | 3 |  |
| *A. prasina* | CESS340 | 194 | 174 | 16 | 6 | 2 | 0 | 18 | 4 | - | 0 |  |
| *A. prasina* | CESS347 | 200 | 178 | 16 | 6 | 4 | 0 | 18 | 4 | - | 0 |  |
| *Dendrelaphis sp.* | CESS004 | 169 | - | 18 | 5 | 2 | 0 | 20 | 5 | 15 | 0 | 20-34 |
| *D.* cf. *pictus* | CESS163 | 182 | - | 18 | 6 | 2 | 0 | 22 | 4 | - | 0 |  |
| *D.* cf*. tristis* | CESS168 | - | - | 18 | 4 | 2 | 0 | 20 | 4 | - | 0 |  |
| *D.* cf. *ashoki* | CESS169 | 178 | 165 | 18 | 6 | - | 0 | 20 | 4 | - | 0 |  |
| *D. chairecaeos* | CESS416 | 183 | - | 18 | 4 | - | 0 | 20 | 4 | 15 | 0 |  |
| *D. tristis* | CESS577 | 185 | 136 | 18 | 4 | 2 | 0 | 20 | 4 | 15 | 0 |  |
| *D. tristis* | CESS113 | 182 | 132 | 18 | 4 | 2 | 0 | 20 | 4 | - | 0 |  |
| *D. cf girii* | CESS442 | 173 | 153 | 18 | 4 | 2 | 0 | 22 | 4 | - | 0 |  |
| *D. cyanochloris* | CESS236 | 188 | 168 | 18 | 6 | 2 | 0 | 20 | 4 | - | 0 |  |
| *D. tristis* | CESS148 | 187 | - | 18 | 6 | 2 | 0 | 20 | 4 | - | 0 |  |
| *Chrysopelea taprobanica** | [1] | 201 | 106 | 18 | 6 | 2 | 0 | - | 4 | 17 | 0 | 20-22 |
| *C. cf. taprobanica** | [2] | 202 | 106 | 18 | 6 | 2 | 0 | 16 | 4 | 17 | 0 |  |
| *C.taprobanica** | [3] | 208 | 120 | 18 | 6 | 2 | 0 | - | 4 | 17 | 0 |  |
| *C. ornata** | [4] | 204-236 | 118-138 | 18 | 6 | 2 | 0 | - | 4 | 17 | 0 |  |
| *Dryophiops philippina** | [5] | 177-184 | 118-123 | - | - | - | 0 | - | - | 15 | 0 | 20 |
| *Dryophiops rubescens** | [5] | 188-199 | 111-136 | 18 | 6 | - | 0 | - | 4-6 | 15 | 0 |  |

**^@^** Values from both left and right side are added for SL, SL2, L, PRSO, IL and PO.

* Data extracted from literature [1-5].

**References**

1. Guptha B, Prasad NVS, Maddock ST, Deepak V. First record of *Chrysopelea taprobanica* Smith, 1943 (Squamata: Colubridae) from India. Check List. 2015;11: 1523.

2. Narayanan S, Joseph N, Kumar R, Vengatesan A. Occurrence of the Sri Lankan Flying Snake, *Chrysopelea* cf. *taprobanica* (Smith 1943) in Tamil Nadu, India.. IRCF REPTILES & AMPHIBIANS. 2017 Apr;24(1):58–60.

3. Constable JD. Reptiles from the Indian Peninsula in the Museum of Comparative Zoology. Bulletin of the Museum of Comparative Zoology*.* 1949*;*103: 59–160.

4. Boulenger, G.A.The Fauna of British India, including Ceylon and Burma: Reptilia and Batrachia. Taylor and Francis, London, 1890:371.

5. Boulenger, G.A. 1896. Catalogue of the Snakes in the British Museum (Natural History). containing the Colubridæ (Opisthoglyphae and Proteroglyphae), Amblycephalidae, and Viperidae. London: British Museum of Natural History. 1896; III:382.
