# Supplementary material for: Discovery of a deeply divergent new lineage of vine snake (Colubridae: Ahaetuliinae: Proahaetulla gen. nov.) from the southern Western Ghats of Peninsular India with a revised key for Ahaetuliinae
Source: PLoS One. 2019 Jul 17;14(7):e0218851. doi: 10.1371/journal.pone.0218851 (PMC6636718; doi:10.1371/journal.pone.0218851)
Supplement: S3 Table — (DOCX) [file pone.0218851.s005.docx]

**Table S3.** **List of external fossil records and secondary calibration used in this study to estimate the time of divergence of *Proahaetulla* gen nov. and Ahaetuliinae.**

| **Calibrations** | **Age range (95% HPD)** | **Tree prior distribution** | **References** |
| --- | --- | --- | --- |
| tmrca Viperidae | 43.18 -61.64 Ma | normal | [1] |
| tmrca Colubridae | 33-65 Ma | normal | [2, 3] |
| tmrca *Zamenis* | 6.0-20 Ma | normal | [4] |
| tmrca Colubroidea | 48.6-95 Ma | normal | [5, 6] |

*We used secondary calibration from Alencar *et al.* 2016 (Age and HPD obtained from dataset 2).

**References**

1. Alencar LRV, Quental TB, Grazziotin FG, Alfaro ML, Martins M, Venzon M, Zaher H. Diversification in vipers: Phylogenetic relationships, time of divergence and shifts in speciation rates. Mol Phylo et Evol. 2016;105: 50–62.
2. Holman JA. Fossil Snakes of North America: Origin, Evolution, Distribution, Paleoecology. Bloomington: Indiana University Press. 2000.
3. Pyron RA, Burbrink FT. Extinction, ecological opportunity, and the origins of global snake diversity. Evolution. 2012;66:163–178.
4. Burbrink FT, Lawson R. How and when did Old World ratsnakes disperse into the New World? Molecular Phylogenetics and Evolution. 2007;43(1):173-189.
5. Rage JC, Folie A, Rana RS, Singh H, Rose KD, Smith T. A diverse snake fauna from the early Eocene of Vastan Lignite Mine, Gujarat, India. Acta Palaentologica Polonica. 2008;53(3):391–403.
6. Chen X, Huang S, Guo P, Colli GR, Montes de Oca AN, Vitt LJ, Pyron RA, Burbrink FT. Understanding the formation of ancient intertropical disjunct distributions using Asian and Neotropical hinged-teeth snakes (*Sibynophis* and *Scaphiodontophis*: Serpentes: Colubridae). Mol Phylogenet Evol. 2013;66(1):254-261.
