# Supplementary material for: Discovery of a deeply divergent new lineage of vine snake (Colubridae: Ahaetuliinae: Proahaetulla gen. nov.) from the southern Western Ghats of Peninsular India with a revised key for Ahaetuliinae
Source: PLoS One. 2019 Jul 17;14(7):e0218851. doi: 10.1371/journal.pone.0218851 (PMC6636718; doi:10.1371/journal.pone.0218851)
Supplement: S4 Table — (DOCX) [file pone.0218851.s006.docx]

**Table S4**. **Details of PCA summary, loadings and scores.**

**Fig 3A**: PCA of *Ahaetulla* members including all species of Peninsular India.

SUMMARY

| **PC** | **1** | **2** | **3** | **4** | **5** | **6** | **7** | **8** | **9** | **10** |
| --- | --- | --- | --- | --- | --- | --- | --- | --- | --- | --- |
| **Eigenvalue** | 1283.13 | 59.22 | 7.99 | 3.70 | 0.970 | 0.594 | 0.152 | 0.076 | 0.00 | 0.00 |
| **Variance (%)** | 94.64 | 4.37 | 0.59 | 0.273 | 0.072 | 0.044 | 0.011 | 0.006 | 0.00 | 0.00 |

LOADINGS

| **PC** | **1** | **2** | **3** | **4** | **5** | **6** | **7** | **8** | **9** | **10** |
| --- | --- | --- | --- | --- | --- | --- | --- | --- | --- | --- |
| V | 0.572 | 0.718 | -0.380 | 0.075 | -0.035 | -0.075 | 0.003 | -0.005 | 0.000 | 0.000 |
| C | 0.818 | -0.527 | 0.219 | -0.029 | 0.012 | 0.058 | 0.008 | -0.006 | 0.000 | 0.000 |
| SL | -0.005 | -0.044 | -0.114 | -0.044 | -0.013 | 0.086 | -0.350 | -0.387 | 0.838 | 0.043 |
| SL2 | 0.018 | 0.068 | 0.026 | -0.614 | -0.062 | 0.036 | -0.141 | 0.449 | 0.088 | 0.618 |
| L | 0.022 | 0.111 | 0.179 | -0.308 | 0.810 | -0.338 | 0.144 | -0.264 | 0.000 | 0.000 |
| PRSO | -0.018 | -0.147 | -0.181 | 0.657 | 0.292 | -0.117 | 0.060 | 0.126 | 0.070 | 0.625 |
| IL | -0.009 | 0.019 | -0.190 | -0.062 | 0.328 | 0.878 | 0.284 | -0.011 | 0.000 | 0.000 |
| PO | 0.012 | 0.035 | 0.063 | 0.166 | 0.360 | 0.048 | -0.461 | 0.656 | 0.151 | -0.412 |
| Mscale | -0.005 | -0.037 | -0.033 | -0.026 | -0.094 | -0.205 | 0.732 | 0.363 | 0.494 | -0.185 |
| Keels | 0.033 | 0.404 | 0.829 | 0.237 | -0.086 | 0.198 | 0.067 | -0.021 | 0.136 | 0.147 |

SCORES

| **PC** | **1** | **2** | **3** | **4** | **5** | **6** | **7** | **8** | **9** | **10** |
| --- | --- | --- | --- | --- | --- | --- | --- | --- | --- | --- |
| *P. antiqua* sp. nov. | 29.25 | 11.39 | 5.70 | 0.564 | -0.226 | -0.846 | 0.810 | 0.335 | 0.000 | 0.000 |
| *P. antiqua* sp. nov. | 39.64 | 16.75 | 2.25 | 1.30 | 0.643 | 0.921 | -0.824 | -0.228 | 0.000 | 0.000 |
| *A. dispar* | -49.511 | -4.168 | 1.97 | -0.038 | 0.369 | 0.278 | -0.337 | 0.426 | 0.000 | 0.000 |
| *A. dispar* | -37.892 | -5.130 | 1.88 | 0.120 | 0.278 | 0.409 | -0.238 | 0.335 | 0.000 | 0.000 |
| *A. dispar* | -36.869 | 0.982 | -0.693 | 0.042 | 1.66 | -0.889 | 0.042 | -0.205 | 0.000 | 0.000 |
| *A. dispar* | -23.448 | -5.488 | 1.83 | -0.245 | 1.76 | -0.168 | 0.169 | -0.307 | 0.000 | 0.000 |
| *A.* cf. *nasuta* | 17.50 | -7.113 | 0.191 | 1.10 | -0.262 | 0.760 | 0.224 | -0.102 | 0.000 | 0.000 |
| *A.* cf*. nasuta* | -8.438 | -2.363 | -0.890 | 0.978 | -0.159 | 0.189 | -0.002 | 0.100 | 0.000 | 0.000 |
| *A.* cf*. nasuta* | -16.867 | 4.15 | -3.679 | 1.37 | -0.321 | -0.525 | -0.088 | 0.162 | 0.000 | 0.000 |
| *A.* cf. *nasuta* | 3.00 | 1.17 | -3.534 | 1.40 | 0.232 | 1.60 | 0.652 | -0.015 | 0.000 | 0.000 |
| *A.* cf. *nasuta* | 10.97 | -2.934 | -1.181 | 1.46 | -1.011 | -1.462 | -0.409 | -0.031 | 0.000 | 0.000 |
| *A.* cf. *nasuta* | 5.30 | 4.00 | -4.671 | 1.82 | -0.563 | -0.453 | 0.095 | -0.013 | 0.000 | 0.000 |
| *A.* cf*. pulverulenta* | 47.77 | -15.781 | 3.35 | 1.10 | -0.917 | -0.003 | -0.073 | -0.313 | 0.000 | 0.000 |
| *A. perroteti* | -65.450 | 7.00 | 1.33 | -3.035 | -2.086 | 0.286 | 0.105 | -0.436 | 0.000 | 0.000 |
| *A. prasina* | 39.13 | -2.441 | -1.404 | -3.830 | -0.426 | 0.395 | -0.232 | 0.438 | 0.000 | 0.000 |
| *A. prasina* | 45.88 | -0.018 | -2.447 | -4.112 | 1.03 | -0.497 | 0.105 | -0.145 | 0.000 | 0.000 |

**Fig 3B**: PCA of Ahaetuliinae members.

SUMMARY

| **PC** | **1** | **2** | **3** | **4** | **5** | **6** | **7** | **8** | **9** | **10** |
| --- | --- | --- | --- | --- | --- | --- | --- | --- | --- | --- |
| **Eigenvalue** | 718.86 | 280.34 | 11.73 | 9.13 | 4.07 | 0.492 | 0.377 | 0.211 | 0.153 | 0.055 |
| **Variance (%)** | 70.11 | 27.34 | 1.14 | 0.890 | 0.397 | 0.048 | 0.037 | 0.021 | 0.015 | 0.005 |

LOADINGS

| **PC** | **1** | **2** | **3** | **4** | **5** | **6** | **7** | **8** | **9** | **10** |
| --- | --- | --- | --- | --- | --- | --- | --- | --- | --- | --- |
| V | 0.572 | 0.812 | 0.047 | -0.005 | -0.104 | -0.004 | -0.003 | -0.014 | -0.019 | 0.007 |
| C | 0.819 | -0.569 | -0.043 | -0.038 | 0.038 | -0.009 | -0.002 | 0.001 | 0.016 | 0.001 |
| SL | 0.001 | 0.029 | 0.175 | -0.164 | 0.258 | -0.064 | -0.676 | 0.390 | 0.324 | 0.395 |
| SL2 | 0.023 | 0.055 | 0.122 | 0.001 | 0.565 | -0.097 | 0.526 | 0.563 | -0.228 | 0.090 |
| L | 0.020 | 0.022 | 0.004 | 0.191 | 0.268 | 0.885 | 0.061 | -0.182 | 0.092 | 0.251 |
| PRSO | -0.021 | -0.055 | -0.156 | -0.176 | -0.678 | 0.237 | 0.223 | 0.510 | 0.025 | 0.338 |
| IL | 0.012 | -0.090 | 0.907 | 0.315 | -0.254 | 0.006 | 0.069 | 0.015 | 0.025 | -0.015 |
| PO | 0.010 | 0.001 | -0.030 | 0.077 | -0.037 | 0.336 | -0.322 | 0.451 | -0.167 | -0.737 |
| Mscale | -0.002 | 0.031 | 0.006 | -0.126 | 0.049 | 0.001 | 0.319 | 0.056 | 0.879 | -0.320 |
| Keels | 0.025 | 0.007 | -0.321 | 0.885 | -0.044 | -0.187 | -0.046 | 0.175 | 0.177 | 0.110 |

SCORES

| **PC** | **1** | **2** | **3** | **4** | **5** | **6** | **7** | **8** | **9** | **10** |
| --- | --- | --- | --- | --- | --- | --- | --- | --- | --- | --- |
| *P. antiqua* | 26.31 | -0.241 | -5.960 | 9.64 | 0.121 | 0.039 | 0.635 | -0.156 | 0.878 | -0.208 |
| *P. antiqua* | 36.73 | 5.63 | -3.714 | 10.17 | -1.216 | 0.239 | -0.912 | 0.452 | -0.799 | 0.139 |
| *A. dispar* | -52.322 | -13.069 | -0.911 | 0.235 | -0.661 | 0.143 | 0.215 | 0.488 | -0.064 | -0.275 |
| *A. dispar* | -40.700 | -13.891 | -1.063 | -0.177 | -0.907 | 0.033 | 0.171 | 0.409 | -0.019 | -0.229 |
| *A. dispar* | -39.686 | -7.266 | -0.642 | 0.286 | -1.104 | 1.81 | 0.279 | -0.040 | 0.007 | 0.313 |
| *A. dispar* | -26.258 | -14.182 | -1.200 | -0.293 | -0.749 | 1.67 | 0.237 | -0.060 | 0.205 | 0.335 |
| *A.* cf*. nasuta* | 14.70 | -14.907 | -1.605 | -2.088 | -2.392 | -0.484 | -0.044 | -0.003 | 0.128 | 0.010 |
| *A.* cf*. nasuta* | -11.246 | -10.175 | -1.079 | -1.119 | -2.153 | -0.233 | 0.050 | 0.139 | -0.044 | -0.079 |
| *A.* cf*. nasuta* | -19.683 | -3.100 | -0.555 | -0.708 | -2.669 | -0.140 | 0.073 | 0.119 | -0.235 | -0.078 |
| *A.* cf*. nasuta* | 0.229 | -6.220 | 0.900 | -0.809 | -3.434 | -0.318 | 0.138 | 0.033 | -0.070 | -0.036 |
| *A.* cf. *nasuta* | 8.13 | -10.173 | -3.071 | -2.415 | -2.184 | -0.427 | -0.164 | -0.038 | -0.047 | 0.036 |
| *A.* cf. *nasuta* | 2.49 | -2.792 | -0.726 | -1.461 | -3.340 | -0.346 | -0.014 | -0.054 | -0.194 | 0.022 |
| *A.* cf. *pulverulenta* | 44.96 | -23.688 | -4.251 | -3.909 | -1.794 | -0.797 | -0.289 | -0.158 | 0.363 | 0.125 |
| *A. perroteti* | -68.269 | -2.717 | -0.166 | 4.17 | 2.51 | -2.063 | 0.93 | -0.801 | -0.034 | 0.339 |
| *A. prasina* | 36.35 | -9.964 | -0.382 | -1.685 | 2.04 | -0.240 | 1.28 | -0.359 | -0.532 | -0.448 |
| *A. prasina* | 43.10 | -7.325 | -0.265 | -1.487 | 2.10 | 1.47 | 1.37 | -0.804 | -0.396 | 0.098 |
| *Dendrelaphis* sp. | -7.082 | -10.184 | 2.00 | 0.211 | 2.68 | 0.481 | -0.696 | 0.660 | -0.096 | -0.623 |
| *D.* cf. *pictus* | 0.387 | 0.255 | 4.58 | 0.661 | 1.44 | 0.014 | 0.329 | 0.634 | -0.119 | 0.181 |
| *D.* cf*. tristis* | 0.041 | -0.067 | 2.50 | 0.031 | 0.871 | 0.197 | -0.859 | -0.516 | 0.296 | 0.028 |
| *D.* cf. *ashoki* | 19.85 | -17.965 | 1.42 | -1.049 | 3.23 | -0.660 | 0.110 | 0.764 | 0.276 | 0.071 |
| *D.* cf. *chairecaeos* | 0.880 | 1.12 | 2.56 | -0.039 | 0.571 | -0.251 | -0.978 | -0.460 | -0.006 | -0.004 |
| *D. tristis* | 0.062 | 4.12 | 2.76 | 0.136 | 0.407 | 0.205 | -0.949 | -0.581 | -0.035 | 0.135 |
| *D. tristis* | -4.930 | 3.97 | 2.80 | 0.271 | 0.581 | 0.251 | -0.846 | -0.526 | 0.187 | 0.028 |
| *D. cyanochloris* | 28.03 | -11.542 | 1.76 | -1.122 | 2.44 | -0.280 | 0.099 | 0.534 | 0.184 | 0.267 |
| *D. tristis* | 3.22 | 4.45 | 3.00 | 0.004 | 1.43 | -0.017 | 0.173 | 0.532 | -0.261 | 0.245 |
| *D.* cf. *girii* | 7.15 | -15.471 | 3.28 | 0.156 | 1.79 | 0.112 | -0.726 | -0.357 | 0.732 | -0.053 |
| *C. taprobanica* | -15.551 | 36.16 | -13.062 | -5.367 | 3.93 | 0.090 | -0.630 | 0.106 | 0.007 | 0.066 |
| *C.* cf. *taprobanica* | -14.791 | 35.54 | 1.50 | -0.331 | -0.235 | 0.189 | 0.476 | 0.339 | 0.385 | -0.165 |
| *C. taprobanica* | 0.129 | 32.26 | 3.00 | -0.263 | -0.839 | 0.057 | 0.561 | 0.293 | 0.542 | -0.146 |
| *C. ornata* | 30.88 | 44.75 | 3.51 | -1.098 | -3.063 | -0.204 | 0.422 | -0.091 | 0.306 | 0.057 |
| *D. philippina* | -11.189 | 10.85 | 1.27 | 0.098 | 0.121 | -0.042 | -0.324 | -0.907 | -0.700 | -0.466 |
| *D. rubescens* | 8.08 | 15.77 | 1.85 | -0.664 | 0.462 | -0.494 | -0.115 | 0.407 | -0.845 | 0.315 |
